# Molecular pathways associated with the nutritional programming of plant-based diet acceptance in rainbow trout following an early feeding exposure

**DOI:** 10.1186/s12864-016-2804-1

**Published:** 2016-06-13

**Authors:** Mukundh N. Balasubramanian, Stephane Panserat, Mathilde Dupont-Nivet, Edwige Quillet, Jerome Montfort, Aurelie Le Cam, Francoise Medale, Sadasivam J. Kaushik, Inge Geurden

**Affiliations:** INRA, UR1067 NUMEA Nutrition, Métabolisme et Aquaculture, Pôle d’Hydrobiologie INRA, 64310 Saint Pée-sur-Nivelle, France; INRA, UMR1313 GABI Génétique Animale et Biologie Intégrative, 78350 Jouy-en-Josas, France; INRA, UR 1037 Laboratoire de Physiologie et Génomique des Poissons (LPGP), Rennes, France

## Abstract

**Background:**

The achievement of sustainable feeding practices in aquaculture by reducing the reliance on wild-captured fish, via replacement of fish-based feed with plant-based feed, is impeded by the poor growth response seen in fish fed high levels of plant ingredients. Our recent strategy to nutritionally program rainbow trout by early short-term exposure to a plant-based (V) diet versus a control fish-based (M) diet at the first-feeding fry stage when the trout fry start to consume exogenous feed, resulted in remarkable improvements in feed intake, growth and feed utilization when the same fish were challenged with the diet V (V-challenge) at the juvenile stage, several months following initial exposure. We employed microarray expression analysis at the first-feeding and juvenile stages to deduce the mechanisms associated with the nutritional programming of plant-based feed acceptance in trout.

**Results:**

Transcriptomic analysis was performed on rainbow trout whole fry after 3 weeks exposure to either diet V or diet M at the first feeding stage (3-week), and in the whole brain and liver of juvenile trout after a 25 day V-challenge, using a rainbow trout custom oligonucleotide microarray. Overall, 1787 (3-week + Brain) and 924 (3-week + Liver) mRNA probes were affected by the early-feeding exposure. Gene ontology and pathway analysis of the corresponding genes revealed that nutritional programming affects pathways of sensory perception, synaptic transmission, cognitive processes and neuroendocrine peptides in the brain; whereas in the liver, pathways mediating intermediary metabolism, xenobiotic metabolism, proteolysis, and cytoskeletal regulation of cell cycle are affected. These results suggest that the nutritionally programmed enhanced acceptance of a plant-based feed in rainbow trout is driven by probable acquisition of flavour and feed preferences, and reduced sensitivity to changes in hepatic metabolic and stress pathways.

**Conclusions:**

This study outlines the molecular mechanisms in trout brain and liver that accompany the nutritional programming of plant-based diet acceptance in trout, reinforces the notion of the first-feeding stage in oviparous fish as a critical window for nutritional programming, and provides support for utilizing this strategy to achieve improvements in sustainability of feeding practices in aquaculture.

**Electronic supplementary material:**

The online version of this article (doi:10.1186/s12864-016-2804-1) contains supplementary material, which is available to authorized users.

## Background

Achieving independence on wild fish-derived feed inputs is a priority for the aquaculture industry to ensure its further sustainable growth [1]. In this respect, the reduced growth performance seen in farmed fish, including rainbow trout, when fed high levels of plant-based ingredients in lieu of fishery-derived fish meal and fish oil is a problem [2, 3]. Despite meeting all known nutrient requirements [4], the poor feed intake and utilisation efficiency of terrestrial plant-based ingredients in fish, including salmon and trout, is not fully understood. In order to analyse the metabolic consequences of partial or complete substitution of fishmeal and fish oil with plant-based sources, studies based on nutrigenomic approaches have been undertaken, comparing hepatic transcriptome changes in trout fed fishmeal or fish oil-free diets [5], total replacement of fishmeal or fish oil with plant sources in trout [6] and European sea bass [7], fishmeal substitution with plant proteins in Atlantic salmon [8], and a proteomic study on the effect of plant-protein substitution on hepatic metabolism in trout [9]. Taken together, these studies showed that plant-feeding altered the expression of actors involved in a large variety of biological functions. Low acceptance, in terms of food intake and also nutrient utilisation, is often attributed to the presence of various plant secondary metabolites that can act as anti-nutritional factors (ANFs) [10, 11]. Examples of the lack of adaptation of rainbow trout to purified ANFs concern alkaloids found in legumes like pea and lupins [12, 13] and saponins found in soybean [14]. Strategies to overcome the poor acceptance of plant-based ingredients in trout have included the use of selective breeding techniques to select for fish with a greater ability to grow on plant-based feeds [15–17].

In mammals, it is widely accepted that nutritional intervention at critical periods of early development, such as during gestation of the foetus or lactation of neonates, can have life-long impact on the organism’s physiology and metabolism, long after the original nutritional intervention, a phenomenon known as nutritional programming [18–21]. Nutritional programming is believed to confer adaptive advantages to the organism to better sustain itself later in life when the adult nutritional environment corresponds with that encountered during early development. The metabolic and physiological changes induced by nutritional programming in mammals are often accompanied by changes in protein or mRNA levels or, in some cases, persistent epigenetic changes in DNA methylation or histone modifications at regulatory enhancer and promoter regions of candidate genes [22, 23]. Such persistent modifications can even lead to trans-generational metabolic changes as shown from the Dutch famine study on starvation-induced nutritional programming during World War II [24]. Some recent studies in fish also report long-term metabolic modifications, albeit at the molecular level, due to early nutritional intervention at the first-feeding stage, such as high carbohydrate intake [25, 26] or changed dietary fatty acid profiles [27, 28]. The results of the above studies validate the first-feeding stage as an effective nutritional programming window. Instead, only few long-term effects were noted in zebrafish following injection of glucose at the embryo stage, directly into the egg [29]. In addition to the nutritional programming of metabolic pathways, studies in mammalian models suggest the ability of early flavour experiences to drive adult flavour and feeding preferences in humans [30]. Similarly, studies on conditioned flavour aversion in rats indicate that the early exposure to either pleasant or noxious flavours may affect adult behaviour through the programming of sensory and cognitive systems [31–33].

**Fig. 1:**
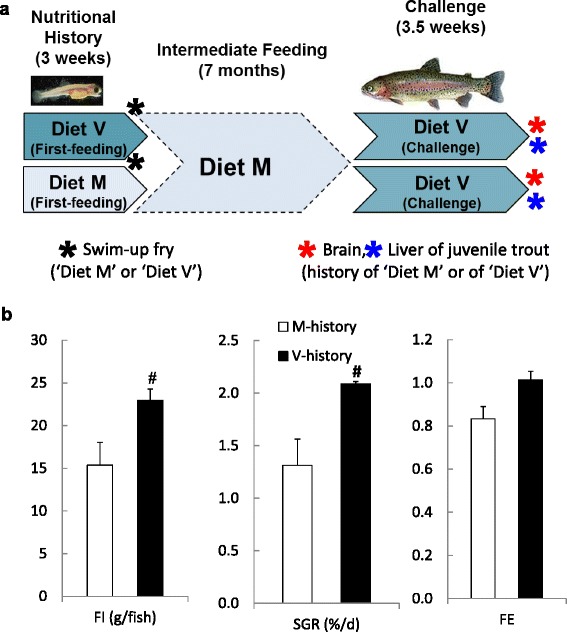
Experimental design evaluating impact of early-feeding exposure of plant-based diet on its future acceptance. **a** Feeding protocol. Rainbow trout swim-up fry (50–150 mg) received for 3 weeks either the plant-based diet V or diet M (first-feeding period, details on diet are given in text) after which both groups received diet M (7-month intermediate growth phase). Both groups were then challenged for 25 days by feeding diet V during which voluntary feed intake, growth and feed utilisation efficiency were monitored (V-challenge. No difference in initial body weight was noted at the start of the V-challenge. The asterix indicate the sampling times for the present microarray study. **b** During the final V-challenge, fish of nutritional history V vs M displayed a higher (*P* < 0.05) feed intake (FI) and specific growth rate (SGR) and a tendency for improved efficiency of feed utilization (FE, *P* = 0.06), underlining the positive impact of the early diet V feeding (see [34] for extra details on the rearing conditions and V-challenge results). # denotes a significant difference due to nutritional history (*P* < 0.05)

We recently attempted to exploit the phenomenon of nutritional programming to improve the acceptance of plant-based feed in rainbow trout [34]. Two groups of trout swim-up fry transitioning to exogenous feeding were exposed to either a control fish meal and oil diet (M-diet) or a plant-based diet (V-diet) for three weeks. Both groups were maintained on the control diet M for 7 months. When the two groups of juvenile trout were then challenged for 25 days with the plant-based V diet, we observed a significantly higher feed intake, growth rate and feed utilization in the V- compared to M-fish (see Fig. 1 and [34]). These long-term positive effects due to the early plant-diet exposure on later plant-diet acceptance suggest that the fish have been nutritionally programmed [34].

To identify the molecular pathways mediating the nutritional programming phenotype observed in our study in trout [34], we utilised microarray expression analysis in order to monitor molecular changes related with the early plant-feeding in 3-week fry fed diet M or diet V and in the liver and brain of juvenile M-fish and V-fish challenged with the same plant-based V diet (Fig. 1a).

## Results and discussion

### Expression profiling

**Fig. 2:**
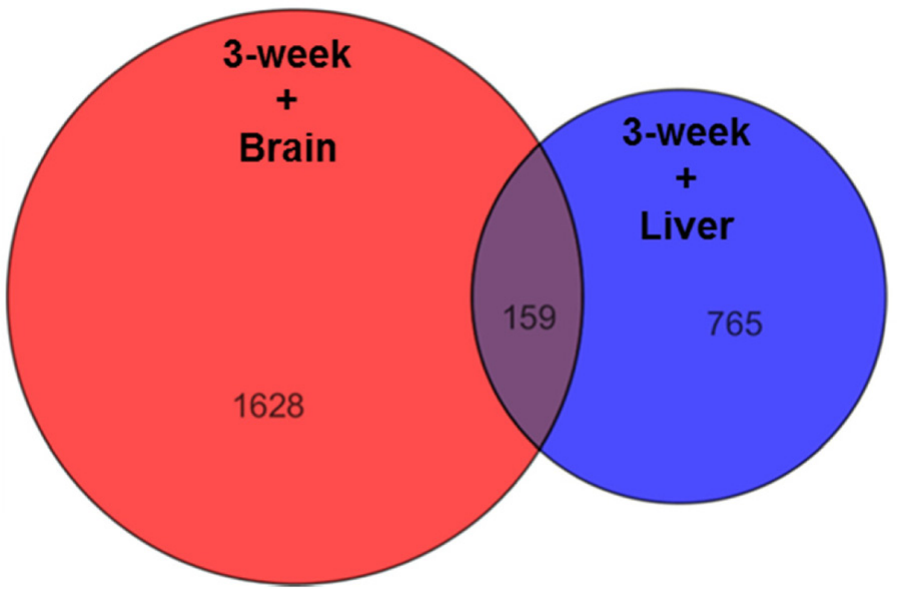
Venn diagram showing differentially expressed mRNA probes by microarray analysis. Summary of number of mRNA probes that are differentially expressed following early nutritional exposure (3-week) in trout swim-up fry, and after V-challenge in the juvenile trout (Brain or Liver). The mRNA probes are all significantly different in expression (p [nutritional history] ≤ 0.05, and fold change ≥ 1.5) in both swim-up fry and juvenile trout (3-week + Brain; 3 week + Liver). The full lists of mRNA probes are outlined in Additional file 1

The present study aims to identify the molecular mechanisms that govern the positive effect of prior plant-diet exposure on the acceptance (feed intake and utilisation efficiency) of the same plant-diet when given 7 months later (see [34]). We therefore performed transcriptomic analysis on swim-up fry collected at the end of early-feeding exposure fed either diet M (fish-based) or diet V (plant-based) as well as brain and liver of juveniles sampled at the end of the V-challenge when both groups received diet V (M-fish and V-fish). The two-way ANOVA performed on differentially expressed probes in whole fry and juvenile brain indicates that 1787 were persistently altered (3-week + Brain; see Additional file 1 and Fig. 2), whereas the 2-way ANOVA of differentially expressed probes in whole fry and juvenile liver showed 924 mRNA probes to be persistently altered by early-feeding exposure (3-week + Liver; see Additional file 1 and Fig. 2). Supervised clustering (as shown in Additional file 2) gives a visual overview of the 1112 up-regulated mRNA probes and the 675 down-regulated mRNA probes in the brain, as well as the 573 up-regulated mRNA probes and the 351 down-regulated mRNA probes in the liver based on nutritional V- vs M-history.

**Fig. 3:**
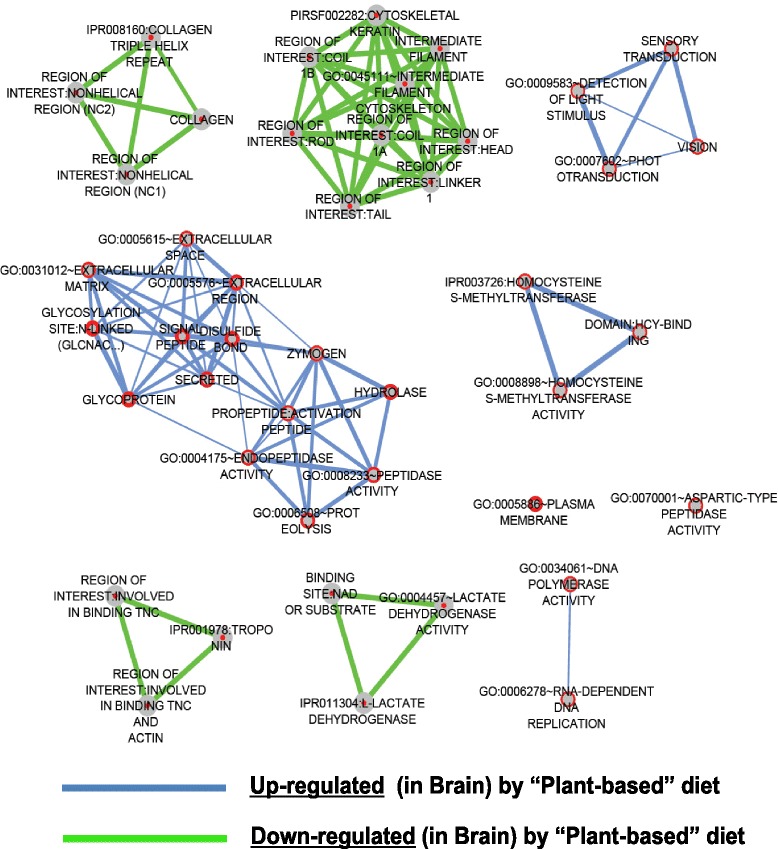
Summary of functional gene ontological analyses significantly enriched based on early nutritional history in the brain. The biological significance of the 1112 up-regulated mRNA probes and 675 down-regulated mRNA probes in the brain of trout based on nutritional history was ascertained by performing gene ontology (GO) analysis using the functional annotation cluster and chart tools from the DAVID (Database for Annotation, Visualization and Integrated Discovery) bioinformatics resource [137, 138]. The resulting data was used as input in the DAVID Enrichment Map plugin from the Bader Lab [139, 140] in the Cytoscape network visualization tool (version 2.8.3) [141–143]. The nodes (circles) represent individual GO terms and the lines represent the relationship between the genes assigned to respective GO terms. Blue and green lines represent up-regulated and down-regulated mRNA probes in the brain respectively

**Fig. 4:**
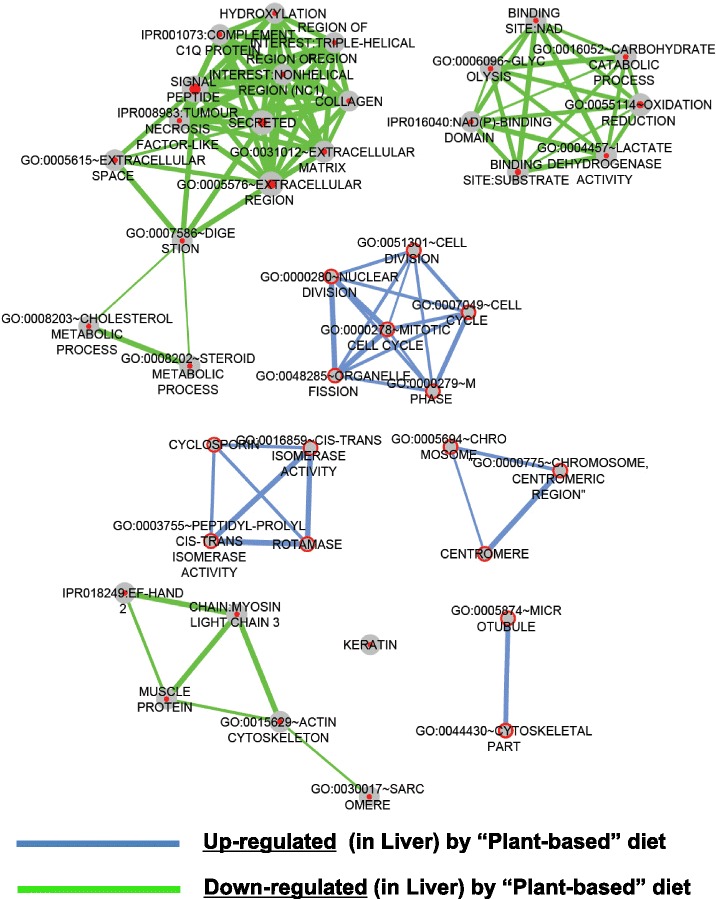
Summary of functional gene ontological analyses significantly enriched based on early nutritional history in the liver. The biological significance of the 573 up-regulated mRNA probes and 351 down-regulated mRNA probes in the liver of trout based on nutritional history was ascertained by performing gene ontology (GO) analysis using the functional annotation cluster and chart tools from the DAVID (Database for Annotation, Visualization and Integrated Discovery) bioinformatics resource [137, 138]. The resulting data was used as input in the DAVID Enrichment Map plugin from the Bader Lab [139, 140] in the Cytoscape network visualization tool (version 2.8.3) [141–143]. The nodes (circles) represent individual GO terms and the lines represent the relationship between the genes assigned to respective GO terms. Blue and green lines represent up-regulated and down-regulated mRNA probes in the liver respectively

**Fig. 5:**
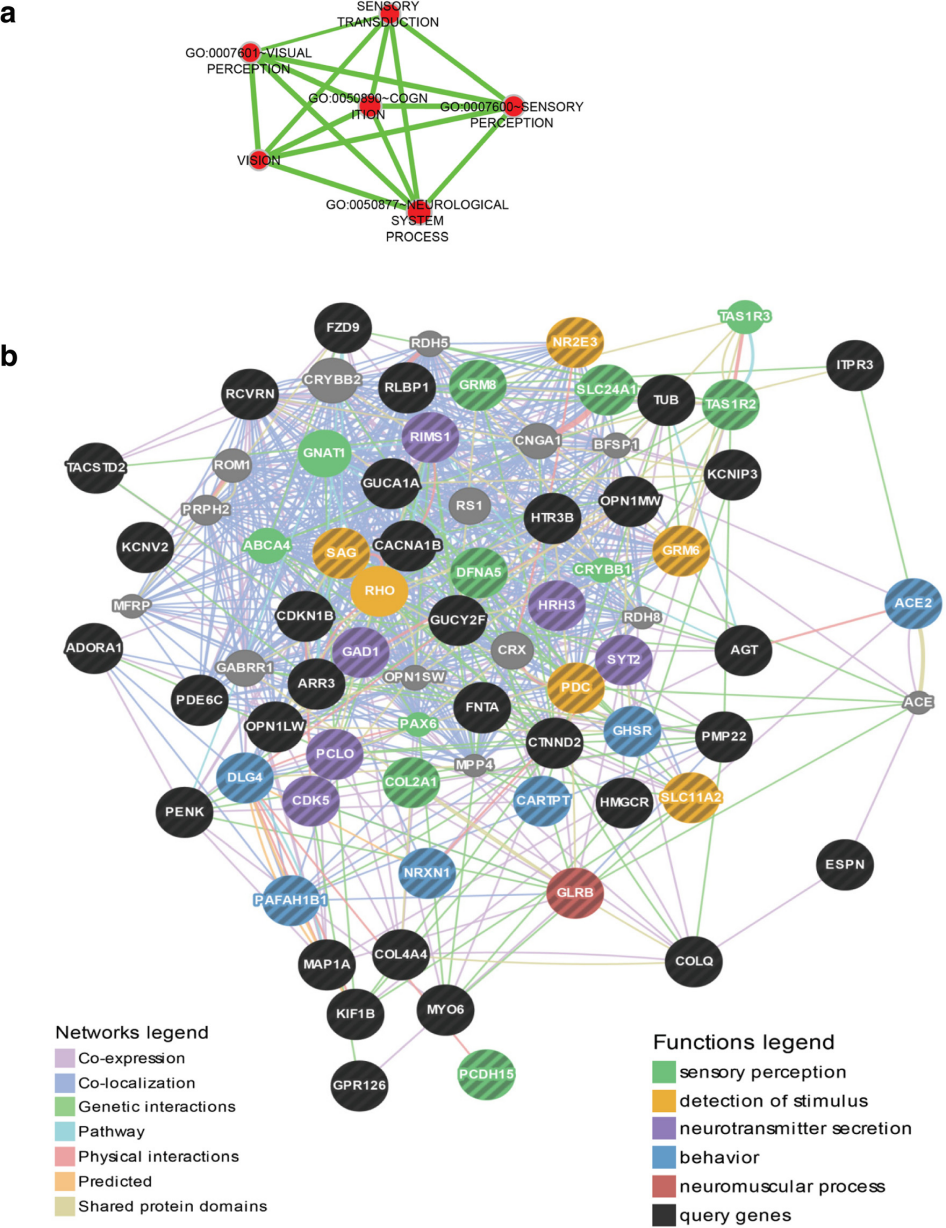
Pathways significantly enriched based on early nutritional history in the brain: Neurological system process. **a** An interconnected cluster of biological processes assigned through GO analysis using DAVID that involve cognition and sensory pathways contributing to neurological system process was identified and visualized as described in Fig. 3. **b** The mRNA probes (see list in Additional file 5) that were assigned to be part of the GO process-neurological system process (represented by nodes with black stripes) were used as input in the GeneMania pathway analysis tool [35–37] to generate networks. The functions legend (nodes) represents the sub-network of the mRNA probes and the network legend (lines) represents the relationship between the genes (see Methods)

The gene ontology analysis, performed to ascertain the biological significance of the differentially expressed mRNA probes, revealed that the genes that are responsive to prior plant-feeding in the brain and liver belong to distinct and interconnected functional categories (Figs. 3 and 4). We acknowledge that the correlation between mRNA expression in whole trout fry (3-week) and that in the liver or brain in juvenile trout cannot be definite for all genes identified by the microarray analysis. However, by using DAVID to identify networks in which specific subset of the differentially expressed genes contribute to a biological process, followed by application of GeneMania for pathway analysis [35–37], we observed congruity in the relationship of co-expression and co-localization that exists between the genes (see Fig. 5 and Additional files 3 and 4). This strengthens our discussion on the implication of the differential mRNA expression at first-feeding and later in brain or liver of juvenile trout in relation with the observed positively programmed V-fish phenotype.

### Genes responsive to nutritional history in the brain

The brain being the central organ controlling sensory and homeostatic processes is perhaps one of the more sensitive organs in reacting to early changes in quality of available feed. To our knowledge, this study is the first to probe transcriptomic changes in the brain of trout or other carnivorous-like fish in response to feeding plant-based ingredients. Functional annotation analysis of the differentially expressed mRNAs (Fig. 3) revealed persistent effects of the early plant-diet exposure on processes such as cognition (Fig. 5), sensory transduction (Fig. 5), methionine metabolism (Additional file 3) and on several genes encoding neuropeptides and their receptors that regulate central and peripheral feeding responses (Additional file 3).

#### Sensory perception and transduction

Early flavour experiences are important drivers of life-long flavour acceptance in mammals. Transmittance of flavours connected to the diet of the mother either through amniotic fluid to the foetus or via lactation to the neonatal offspring may help the offspring in future dietary choices [30]. In our study, one prominent cluster of genes identified in the juvenile trout brain to be affected by early diet exposure was assigned to the interconnected biological pathways affecting sensory perception, cognition and neurological system processes (Fig. 5 and Additional file 5).

Several genes in this cluster were associated with visual transduction processes (Fig. 5 and Additional file 5) such as photoreceptor components (Nr2e3, Pdc, Sag, arr3, Pcdh15), genes involved in phototransduction (Gucy2F, Pde6c, Kcnv2, Slc24a1) and photosensitive proteins (Opsin, Pinopsin, Recoverin, Retinaldehyde-binding protein, Arrestin). These generally had a higher expression level in the V-fry and later in the brain of the V-fish when challenged again with the plant-based diet relative to M-fish. A functional sense of visual perception contributes to the ability of salmonids to consume various types of pelleted feed [38]. More recently, transcriptomic analysis of brain of hybrid mandarin fish showed differential expression of genes encoding for retinal photosensitivity and cognitive function depending on their feeding habits [39]. Possibly, the early feeding experience modified functional visual acuity affecting the later feeding behaviour in rainbow trout. Other interesting genes involved in sensory perception whose expression showed both short and long-term modification in the trout brain are olfactomedin (OLFM), espin (ESPN) and a member of the taste receptor family (Tas1r2) (Fig. 5 and Additional file 5). Tas1r2 (also known as T1R2) constitutes one-half of the heterodimeric taste receptor involved in recognition of sweet taste [40]. In our study, brain Tas1r2 was upregulated by early exposure to plant-ingredients. Though there appears to be a loss of Tas1r2 in obligate carnivores such as the domestic cat unable to taste sweet substances [41], recent studies in rainbow trout detected extraoral Tas1r2 expression as documented in intestine [42] and in hypothalamus or hindbrain [43]. Tas1r2 expression in the latter study was found to be responsive to changes in glucose levels. Also in mammalian brain, Tas1r2 has been suggested to act as a glucosensor in energy-sensing hypothalamic neurons [44]. In the case of salmonids, gustation is believed to play a minor role in driving feeding preference when compared to olfaction [45]. Additionally, olfactory imprinting is suggested to be the primary mechanism used by adult salmon to return to their natal streams (homing) with free (L-) amino acids assigned as the olfactory cues directing this phenomenon [46–48]. Olfactomedin (Fig. 5 and Additional file 5) is a secreted glycoprotein involved in regulating maintenance or differentiation of chemosensory cilia in olfactory neurons, as well as axonal growth, branching and optic nerve extension [49]. ESPN, also responsive to nutritional programming in our study, is an intriguing candidate in terms of genes that encode structural proteins that mediate sensory perception. ESPN (Fig. 5 and Additional file 5) is a multifunctional actin-bundling protein expressed in brain tissues, retina and the inner ear, mediating sensory transduction in various mechano- and chemosensory cells, important for vertebrates to sense their environment [50]. The observations from studies elaborating on the perception of the umami flavour [51] together with evidences of visual stimuli activating gustatory responses [52] indicate the synergism of primary senses in the formation of flavour preferences. Thus, it appears that early plant-diet exposure in trout may mediate feed acceptance of the same diet at a later life stage by affecting pathways regulating the sensory perception of taste, odour and possibly vision.

#### Cognition and synaptic plasticity

In mammalian models, the exploration of flavour perception for evaluating the reward value of food and its behavioural outcomes, has revealed a remarkable integration of the primary cortex centres of taste, olfaction and vision to the higher learning, reward and decision centres in the brain (reviewed in [53]. Whenever an animal interacts with its surrounding environment, its experience drives learning and the formation of memory to modify its behaviour and better adapt its responses to ever-changing conditions. Synaptic plasticity, the ability to change the strength and stability of synapses dynamically in response to various stimuli, forms the fundamental basis of the complex neuronal functions of learning and memory formation based on prior experience [54]. A multitude of proteins mediating processes of cytoskeletal remodelling, cell adhesion, neurotransmitter production and secretion, receptor function and signal transduction, either pre- or post-synaptically, play an essential role in synaptic plasticity. As part of a larger and connected network of neurological system process, the early plant feeding induced short (following first-feeding) and long-term (during V-challenge) changes on the transcriptomic profile of genes involved in cognitive process (Fig. 5 and Additional file 5) like receptors involved in stimulus detection (SLC11A2, Grm6, Grm8), neurotransmitter secretion (RIMS1, GAD1, PCLO, CDK5, HRH3, Syt2), response to external stimuli (Ghsr, CARTPT, NRXN1, DFNA5, PAFAH1B1, Pafah1b1b, DLG4, Kcnip3) and synaptic transmission and memory formation (COL2A1, Glrb, Dlg4, CTNND2, ADORA1, Gpr126, Itpr3, FZD9).

The maintenance of sustained synaptic plasticity to encode the information and memory of any particular stimulus, including that related to given taste, known as long term potentiation (LTP), often relies on tight regulation of neurotransmitter levels [55]. Neurotransmitter release occurs at the presynaptic active zone (AZ), where pools of synaptic vesicles containing neurotransmitters are maintained. Piccolo (Pclo), a cytoskeletal matrix protein expressed in the brain of mammals [56] and of teleosts [57], is associated with the AZ and is involved in the functioning of glutamine- and γ-amino butyric acid (GABA)-responsive synapses [56]. Pclo knockdown in mice leads to impairment of spatial learning and a decrease in LTP [58], highlighting its role in establishing functional synapses for stimulus-driven memory formation. Like Pclo, regulating synaptic membrane exocytosis protein 1 (RIMS1) is another evolutionary conserved scaffolding protein of AZ that regulates neurotransmitter release [59]. RIMS1 controls the pool of synaptic vesicles and functions to maintain homeostatic synaptic plasticity that is essential for the efficiency of synaptic network activity [54]. A key role in the regulation of the pool of synaptic vesicles during homeostatic plasticity was also suggested recently for cyclin-dependent protein kinase 5 (CDK5) [60] as perturbing the levels of CDK5 by pharmacological inhibition of CDK5 or gene deletion in mice impairs the regulation of synaptic vesicle pools and disrupts homeostatic synaptic plasticity [54]. The persistent changes in the expression of genes that control neurotransmitter release through several distinct mechanisms (Pclo, RIMS1, CDK5) following the early plant-diet exposure suggest that neurotransmitter release may be one process involved in programming the enhanced feed acceptance of plant-based diet in juvenile trout confronted with this diet later in life. Besides neurotransmitter release, enzymes involved in neurotransmitter synthesis might contribute to synaptic adaptation. Dynamic changes in transcript levels of glutamate decarboxylase (GAD1), as observed in this study, control GABA synthesis and regulate the inhibitory GABAergic potentiation at interneurons, important for the functioning of activity-driven synaptic plasticity [61, 62]. Of interest, GAD1 expression has been found to be tightly controlled during gustatory habituation [63]. Furthermore, the activity-dependent epigenetic changes at the GAD1 proximal promoter in olfactory bulb interneurons [64] and the conserved function of GAD1 in teleost forebrain development [65] hint at GAD1 as a target associated with the programming of plant-based diet acceptance in the trout brain. This warrants further detailed exploration.

Early plant-diet exposure was found to exert long-term effects on several other classes of genes mediating synaptic plasticity, in addition to those regulating neurotransmitter levels. Platelet-activating factor acetyl hydrolase IB subunit alpha b (Pafah1b1b), also called Lis1, plays an important role in neuronal migration during development. Its mutation in mammals [66] and in zebrafish [67] causes lissencephaly, a severe brain malformation in which cognitive defects are associated with loss of complexity of the cerebral cortex. Given the role of Pafah1b1b in synaptic transmission in adult neurons [68] and its expression in the neurons of the early sensory system in the teleost embryo [69], the long-term changes in Pafah1b1b expression due to the early plant feeding in brain of trout displaying the nutritional programming phenotype is intriguing. Moreover, in rodents, changes in the lissencephaly-1 gene mRNA levels due to environmental stimulation have been suggested to modify synaptic plasticity and animal learning and memory performance [70]. Catenin delta 2 (CTNND2), a member of conserved p-120 cadherin family [71] is a constituent protein at adherens junctions between neurons where it plays a crucial role in maintenance of synapses and in ensuring stable memory storage [72]. There is also evidence of delta catenins in neuroplastic processes in the peripheral retinal [73] and olfactory pathways [74], which make it an interesting candidate gene for our study. The expression of genes encoding neurotransmitter receptors (Fig. 5 and Additional file 5) was also persistently modified by the early diet exposure including the adenosine receptor A1 (ADORA1). As adenosine, formed from the catabolism of ATP, can be directly released by neurons and glia, activation of ADORA1 modulates synaptic transmission. Adenosine and ADORA1 activation have been reported to be involved in LTP impairments caused by distinct types of prior experience (e.g. stress) susceptible to affect memory formation [75]. In the teleost nervous system, ADORA1 expression is highly affected by stimulation during zebrafish development [76] and has been implicated in processes of anxiety and arousal [77] and of photoreceptor function [78], rendering its association with the present nutritional programming phenotype and cognition noteworthy.

The interpretation of the functional implications regarding ‘how does early diet experience act on the brain to store information and change behaviour’ based on the current microarray analysis in the whole trout brain is challenging, and we are aware that we have possibly overlooked relevant region-specific neuronal processes. However, there is precedence for obtaining useful insights into the molecular mechanisms underlying behavioural responses from whole-brain transcriptomic studies in teleosts [79–83]. Moreover, as discussed earlier, the use of pathway analysis to ascertain co-expression and co-localization patterns for the genes within a network aids in proving greater biological robustness regarding the relevance of the data in relation with the observed phenotype. Certainly, histochemical analysis of selected genes in the fry and juvenile brain [84, 85] would be useful to ascertain the site of action influencing cognition, learning and memory formation regarding plant-diet acceptance in rainbow trout. Despite these concerns, the finding that the early diet exposure induced long-term changes in expression of genes involved in sensory perception, sensory transmission, cognition, memory formation and neuronal development is an exciting observation for further studies on the potential of nutritional programming of plant-based diet acceptance in trout or other teleosts.

#### Homocysteine and methionine metabolism

Several mRNA probes encoding genes involved in homocysteine-methionine metabolism were differentially expressed according to the nutritional history (Additional files 5 and 3). The expression of methionine synthase (MTR), up-regulated in brain of V- compared to M-fish, is involved in the recycling of methionine from homocysteine using 5-methyl tetrahydrofolate (THF) as the one-carbon substrate [86]. Similarly, BHMT and BHMT2 also convert homocysteine to methionine using betaine (N,N,N tri-methyl glycine) as substrate [86]. Thus, the expression of three different genes involved in conversion of homocysteine to methionine was enhanced by the prior plant-feeding. Homocysteine is considered as a risk factor for neurodegenerative diseases and deficiencies in MTR correlate with cognitive impairment [87]. In addition, age-dependent reduction in MTR mRNA in humans may be a source of risk for neurological disorders across the lifespan via their impact on methylation reactions, including epigenetic regulation of gene expression [88] as methionine is required for the generation of SAM (S-adenosylmethionine), the primary methyl donor [86]. Indeed, the relative mRNA levels of Mat1a, which catalyzes conversion of methionine to SAM, was affected by early feeding history, being higher in the brain of V-fish compared to M-fish (Additional files 5 and 3). Mat1a was identified as candidate gene in a study on evolutionary conserved longevity genes impacting human cognitive abilities in elderly cohorts [89]. Thus, the long-term effects of the early plant-diet exposure on the regulation of central homocysteine-methionine metabolism warrants further studies from the perspective of maintenance of cognitive function and regulation of epigenetic enzyme substrates in the brain of nutritionally programmed rainbow trout.

#### Appetite and feeding

The functional annotation analysis identified a number of differentially expressed genes associated with the regulation of feeding behaviour (Additional files 5 and 3), of interest in view of the positive effect of the early plant-diet exposure on feed intake during the V-challenge. This set of mRNA probes include neuropeptide hormones (CCK, bombesin, somatolactin, POMCB CRF2, CARTPT), hormone receptors (LEPR, GHSR, HTR3B), transcription regulators (NR1H3, PPARG, Tub) and regulators of neuropeptide hormone levels (AANAT, MAOA). In terms of expression pattern, MAOA, CRF2 and CARTPT were up-regulated following early plant-diet exposure but down-regulated in the brain of V-fish during V-challenge. In contrast, CCK, bombesin, somatolactin, POMCB, LEPR, GHSR, HTR3B, NR1H3, PPARG, Tub and AANAT, all remained upregulated by the prior plant-diet exposure (Additional files 5 and 3). Regarding neuropeptide transcripts, we hypothesized to find higher levels of anorectic peptides in brain of M-fish during the V-challenge, in line with the higher reluctance of these fish to consume the plant-based diet. However, gene expression of bombesin, pro-opiomelanocortin B (POMCB) or cholecystokinin (CCK), known as feeding-inhibitory in fish [90], was higher in brain of V- instead of M-fish. POMCB encodes the inactive precursor that is post-translationally processed to several biogenically active neuropeptides including adrenocorticotropic hormone (ACTH), melanotropin alpha 2 (α-MSH 2) and melanotropin beta 2 (β-MSH 2), collectively referred to as melanocortins. In Atlantic salmon brain, POMCB expression increases postprandially [91]. The cholestokinine gene CCK-T has been found before to be highly expressed in trout brain [92] and, as for POMC, CCK increases after feeding in Atlantic salmon, in line with its role in mediating satiety in fed condition [91]. In our study, the brain samples in the V-challenge were sampled 8 h postprandially. As such, the higher CCK and POMC mRNA levels in the brain of the V- relative to M-fish possibly signal enhanced satiety resulting from the higher intakes during the V-challenge. They would therefore reflect the downstream response rather than the actual basis of the programmed enhanced feed intake. On the other hand, some other peptides known to exert anorectic effects in teleosts [90] had lower expression in the V-fish brain, as initially hypothesized. This was the case for e.g. corticotropin releasing factor (CRF) and cocaine- and amphetamine-regulated transcript protein (CARTPT), which warrant a more targeted approach in order to confirm their possible role in the positive programming effect on plant-feed acceptance due to early plant feeding.

Besides neuropeptides, enzymes that mediate neuropeptide levels may also affect feeding behaviour [93]. An example of this is arylalkylamine N-acetyltransferase (AANAT), the rate limiting enzyme in melatonin synthesis, whose transcription and enzyme activity are crucial in the central regulation of circadian rhythmicity [94]. Some teleosts including rainbow trout have two AANAT genes [95]. In chum salmon *Oncorhynchus keta*, a close teleost homolog of rainbow trout, ANAAT 1 and −2 mRNA abundance has been found in various regions in the brain [95], which may relate with the various neural and endocrine roles attributed to melatonin in fish [96], including feeding. In rainbow trout, treatments with melatonin altered gene expression of brain neuropeptides involved in feeding [97], whereas feed deprivation altered melatonin production partly regulated by reduced AANAT activity [98]. Accordingly, lower AANAT-2 mRNA was seen in the brain of M-fish, possibly reflecting the lower acceptance of the plant-based diet. Given the propensity of AANAT mRNA to fluctuate with meal timing, it would be interesting in the future to measure via a time course, the rhythmicity of mRNA levels and enzymatic activity of AANAT in order to assess possible effects of nutritional programming on circadian rhythms in trout, as seen in the hypothalamus transcriptome of rat following in utero protein restriction [99].

Somatolactin (SL) is a member of the growth hormone/prolactin family and is highly expressed in rainbow trout pituitary and brain [100] where it plays a role in energy homeostasis. In gilthead seabream, plant-protein was found to lower plasma SL levels coincident with lower feed intake [101]. In our study, SL transcripts were significantly lower in brain of M-fish which consumed less than V-fish when challenged with the plant-based diet. Since increases in ration size were found to enhance plasma SL in fish [102], it remains however unclear whether the observed changes in SL mRNA mediate or result from changes in feed acceptance in our study. Transcript levels of several nutrient-sensitive receptors were also modified (Additional files 5 and 3). Ghrelin receptor or growth hormone secretagogue receptor (GHSR) is an important gene of the somatotropic axis, playing an essential role in energy expenditure and food intake [103]. In mammals, GHSR expression is co-localized in neurons with CCK expression, and its high expression in brain reward circuits suggests a role for GHSR in the induction of feed intake resulting from central administration of ghrelin [104]. This may explain the higher GHSR expression in brain of juvenile V-fish showing a relatively superior feed intake when challenged again with the plant-diet. Leptin receptor (LEPR) is a membrane bound receptor that mediates the leptin-induced suppression of feed intake in specific populations of neurons located in the hypothalamus [105]. Juvenile salmon fed restrictively (40 % of the amount of feed given to the control group) displayed decreased growth along with significantly lower LEPR mRNA in the brain [106]. Along these lines, the lower LEPR expression in brain of M relative to V-fish possibly reflects the lower feed intake of that group during the V-challenge. Nuclear receptor subfamily 1 group H member 3 (Nr1h3) or Liver Xa receptor, is an oxysterol-activated nuclear receptor involved in the transcriptional control of whole body cholesterol homeostasis [107]. In mammalian brain, Nr1h3 plays a role in memory functions and the development of age-dependent neurodegenerative changes [108]. In zebrafish midbrain, endogenous Nr1h3 ligands (cholic acid, epoxycholesterol) have been shown to selectively regulate dopaminergic neurogenesis [109]. In our study, rainbow trout exposed at early life to the plant-diet have higher Nr1h3 expression in the brain (Additional files 5 and 3). It would be interesting to investigate if any diet-related or endogenous oxysterol ligands of Nr1h3 are involved in driving memory or cognitive processes related with the positive plant-diet acceptance phenotype observed in this study. 5-Hydroxytryptamine receptor 3B (HTR3B) is a serotonin receptor involved in neural processes related to cognition and emotion in mammalian brain [110] and in mediating a number of satiation signals [111]. Also in rainbow trout, the serotonergic system plays a role in the control of food intake, as shown by the altered feed intake after pharmacological stimulation of serotonin receptor subfamilies [112] or after pre-treatment with the serotonin precursor tryptophan which counteracts stress-induced anorexia [113]. Thus, higher HTR3B expression in brain of juvenile V-trout exposed to the plant-diet at early life may drive improved feed intake through neural functions of satiety and memory.

When we considered using an early exposure to nutritionally program the acceptance of a plant-based diet in rainbow trout [34], we deliberately targeted the critical first-feeding stage during which the reliance on endogenous yolk as nutrient source is diminishing and swim-up fry begin to consume exogenous feed [114]. This developmental first-feeding period in salmonids is accompanied by synchronized anatomical, physiological and behavioural changes, including plasticity of olfactory and taste responses [115, 116]. The present brain transcriptome reveals a very large number of genes affected by the early plant-feeding. These encode a large variety of proteins that regulate sensory perception, cognitive processes, epigenetic changes and neuropeptides mediating feed intake which align contextually with the observed phenotype of enhanced plant-diet acceptance at the juvenile stage, attributed to the initial plant-diet exposure.

### Genes responsive to nutritional history in the liver

The functional annotation analysis of differentially expressed mRNAs in liver (Fig. 4) reveals that the early plant-diet exposure has both short-term and long-lasting effects on intermediary metabolic processes (Additional file 4), zymogens mediating protein degradation (Additional file 4), protein folding and immunomodulatory activity of peptidyl-prolyl isomerases (Additional file 4) and on cytoskeletal proteins involved in stress response and cell cycle (Additional file 4).

Previous studies have utilized nutrigenomic approaches in order to identify changes in hepatic function due to feeding plant- versus fish-based ingredients [5–9]. These all compared the direct diet effects in naïve unconditioned fish fed the different feed for a relatively long period, though never from the first-feeding fry stage onwards. In contrast, our study compares changes in molecular response at first-feeding (short-term direct diet effect) with those induced by the early feeding at the juvenile stage (indirect long-term nutritional programming effect) when fed the same plant-based diet (V-challenge). Nevertheless, several of the candidate genes and functional pathways identified by the previous nutrigenomic studies were found relevant for discussing our data, in particular regarding responses to plant-feeding in the unconditioned M-fish.

#### Intermediary metabolism

A large number of mRNA probes in the liver found to be affected by the early diet exposure, were members of intermediary metabolic processes. These could be further classified as mediating glucose, alpha-amino acid or steroid metabolic process (Additional files 6 and 4). Some of the mRNAs encoding mediators of glucose metabolism (e.g. Pgam1 and Dlat) were up-regulated by early plant exposure, whereas others (e.g. Ldha, LdhB, Gapdh, Pgam2, Aldoa) were permanently down-regulated by the plant-diet history. The higher Aldoa, Pgam2 and Gapdh expressions in the liver of unconditioned M-fish during V-challenge agree with the overall induction of glycolytic mediators by plant-protein feeding in fish [5–9], as shown for higher aldolase expression in liver of European sea bass [7] or in the hepatic proteome in trout [9] fed plant- versus fish-based ingredients. In our study, the lower Aldoa expression in V- than in M-fish hence may indicate a programming effect due to the early plant-diet exposure giving a reversal in the trend of the hepatic glycolytic response to plant-based feed. Several probes of lactate dehydrogenase (LDHA and LDHB) were also affected by nutritional history, all having lower hepatic transcript levels in V- versus M-fish. The significance of the higher LDH expressions in the liver of M-fish is not clearly apparent in the absence of further data on plasma lactate and hepatic LDH activity. LDH, through regulating the cellular redox potential by modifying NAD^+^/NADH levels, has also a role in oxidative stress response, which is another pathway identified to be affected in liver by the early plant-feeding (Additional files 6 and 4). Several oxidative stress-responsive genes, which include enzymes involved in hepatic carbohydrate and lipid intermediary metabolism, had higher mRNA levels in the unconditioned M-fish, consistent with previous observations of higher cellular oxidative stress in salmon fed plant proteins [8].

A group of mRNAs identified to regulate steroid metabolic processes (Fabp6, Tm7sf2, Apoa4, Fdps, Hmgcs1, Srd5a2, Cel, Cyp27a1, sult1st2, sult1st3) were found to be downregulated due to the prior plant-feeding (Additional files 6 and 4). In general, fish fed diets containing soybean meal show upregulated capacity of cholesterol and bile acid synthesis as illustrated by enhanced expression of FDPS, sterol transport genes (APOA1 and APOB100) [7] or genes involved in bile acid metabolism such as HMGCS1 [117], which was attributed to the compensatory response of liver to the lower cholesterol content in plant-based feed. This is unlikely to explain the differential response in our study since the trout during the V-challenge received the same plant-diet and no difference in plasma cholesterol was noted between M and V fish at the end of the V-challenge (6.4 and 6.3 mmol/L, respectively). Alternatively, a study with liver HepG2 cells reported direct effects of soy-extracts and isoflavones on SREBP-2-regulated genes involved in cholesterol biosynthesis and homeostasis such as HMG CoA synthase (HMGCS) mRNA [118]. As such, the higher expression of genes involved in cholesterol biosynthesis (HMGCS1, Tm7sf2 and FDPS) and transport (APOA4) in liver of the unconditioned M-fish during V-challenge possibly reflects the typical molecular response to soy products, seen in fish without an early-life plant-diet experience. Alternatively, as suggested above for glycolytic markers, the lower hepatic response of cholesterol biosynthetic and transport genes to plant-feeding in juvenile V-fish may reflect an adaptive metabolic process induced by the prior plant-feeding. Xenobiotic sterols such as soy phytoestrogens (e.g. genistein or daidzein) have been postulated as a reason for poor palatability of plant-based feed in fish [10] and may accumulate in the bile of rainbow trout [119]. M-fish also had higher hepatic expression of cytosolic sulfotransferases sult1st2 and sult1st3 than V-fish (Additional files 6 and 4). The respective mammalian homologs SULT1E1 and SULT1A1 are known to mediate the detoxification of genistein and daidzein [120] as also shown in HepG2 liver cells [121]. Similarly, the induction of genes mediating the xenobiotic metabolism of phytoestrogens in the juvenile M-fish may indicate an acute adaptive response to the V-challenge. The other way around, this may also be interpreted as reduced sensitivity of V-fish to the presence of anti-nutritional factors, which possibly contributes to the positive programming effect related to the prior plant-diet experience in the V-fish.

#### Zymogens

A group of mRNAs encoding zymogens (pro-enzymes) possessing peptidase activity (Additional files 6 and 4) was found to be differentially expressed following first exposure to plant-based diet (3-week) and after V-challenge (liver). While zymogens were expressed at either a lower (Mmmp2, CASP3, Cela2a, Prss1, Gzma, CTSK, MEP1A, PRSS7) or higher level (Psmb7, CPB1, Prss2, Ctrb1, Lce, Ctrc, Prss3, F5) in in the V-fish after V-challenge, most of the above mRNAs were down-regulated in the fry following the early plant-diet exposure. Changes in the expression of genes encoding proteolytic activity, including members of proteasome complex, have been documented before in studies on the replacement of fish meal by plant-ingredients in fish, albeit with contradictory trends. For example, feeding a plant-diet increased hepatic gene expression of several proteasome subunit members (PSMB) including PSMB7 in European sea bass [7] and also the hepatic protein levels of PSMB in rainbow trout [9]. In contrast, another study with rainbow trout fed plant-based feed showed reduced abundance of PSMB7 and of other transcripts encoding proteasome subunits [5]. This is in line with the lower expression of PSMB7 in the present fry when fed plant-ingredients and in liver of M-fish during the V-challenge (Additional file 6). Alternatively, the prior plant diet experience enhanced PSMB7 expression in liver of V-fish when confronted again to the plant-diet. These findings do not relate with the notion in trout that growth rate and efficiency of protein utilization are negatively correlated to hepatic proteasome activity [122]. The reason for the inconsistent hepatic responses of proteasome subunit to plant-based diets in fish is not apparent and a concurrent evaluation of effects on protein turnover and the activity state of proteases may be useful in future studies [123]. Additionally, several of the zymogen genes identified here may function in lysosomal autophagy-mediated protein degradation [124], which, by regulating levels of key metabolic enzymes, may play a role in the response of liver to nutritional challenges [125]. Several targets of autophagy in the hepatic lysosome, such as Aldoa, Gapdh, Ldha, Ldhb and Cyp27a1 are also modified in the trout liver by the prior plant-diet exposure (Additional files 6 and 4). Possibly, the expression of lysosomal proteases and enzymes mediating intermediary metabolism is co-regulated in trout by the early plant-diet exposure in order to adapt hepatic metabolism to the plant-diet when presented later, as might be the case in the nutritionally programmed V-fish.

#### Intracellular protein folding

V-fish have higher hepatic expression of several peptidyl prolyl cis-trans isomerases (PPIases) important for intracellular protein folding [126], including cyclophillins (PPIB, PPIG) and FK506 binding proteins (FKBP2, FKBP7, FKBP11) (Additional files 6 and 4). PPIB, involved in protein folding in the endoplasmic reticulum (ER), plays a significant role in protecting cells against ER stress [127]. The differential expression of hepatic PPIase mRNA seen in salmon when switched to a plant-based diet may be related with the presence of anti-nutritional factors, increasing reactive oxygen species [8]. Thus, an increase of the multiple genes encoding peptidyl-prolyl cis-trans isomerase activity, as seen for PPIB in liver of the nutritionally programmed V-fish when challenged again to the plant diet may serve to counteract oxidative and ER stress better than in the unconditioned M-fish.

#### Cytoskeleton

A large cluster of mRNAs encoding cytoskeletal proteins (Additional files 6 and 4) was differentially expressed following the first plant-diet exposure (3-week) and after V-challenge (liver). These groups of mRNAs represent members of the actin, intermediate filament and microtubule cytoskeletal components, as well as regulators of mitosis. The differential expression of genes mediating remodelling of cytoskeleton is consistent with previous observations in rainbow trout [6] where substitution of fish-based by plant-based ingredients altered the expression of actin, actin related protein 2/3 complex (Arpc4), keratin, tubulin alpha chain, kinesin members (KIF2C) and dynein 1 intermediate chain (Dync1i2); all of which were identified to be differentially expressed in the trout liver as a result of the early plant-diet exposure (Additional files 6 and 4). Several genes in this pathway also play a key role in cell cycle regulation (Additional files 6 and 4) and are all expressed at a lower level in M-fish during the V-challenge. Trying to unravel the postnatal consequences of nutrient manipulation during foetal life, a study with rat identified genes encoding cell cycle regulators and cytoskeletal remodelling proteins as key drivers of nutritional programming in rat [128]. Of interest, a number of candidate genes from that study, including Arpc4, Ube2c, actin and tubulin members, are also persistently altered by the early plant-diet exposure in our study (Additional file 6), which may indicate a role of these cytoskeletal components in the programmed V-fish phenotype, though the underlying mechanisms remain to be elucidated. Also, it cannot be excluded that these changes are the consequence of the faster growth in the V- versus M-fish during the V-challenge phase.

### Confirmation of selected microarray expressed targets by real-time PCR

Real time PCR analysis (Additional file 7) was performed on selected genes that represent the different pathways (see Additional files 1, 5 and 6) enriched by nutritional history in the brain or liver in order to confirm the significance of differential mRNA expression pattern observed in the microarray data. For all genes tested, despite some differences in the scale of differential expression, the expression pattern between microarray data and real time PCR data was congruent (Additional file 7).

### Nutritional programming of plant-based diet acceptance in teleosts

The magnitude of fold-change difference in transcript levels was generally higher in the 3-week fry than in juvenile trout liver and brain. This is to be ascribed to the fact that the applied start-feeding stage is particularly sensitive to changes in the environment, and also in the setting of exogenous feed preferences in oviparous fish [114, 129], which translates into a more dynamic transcriptomic response than at the later stage, in the juvenile trout. Also, importantly, the molecular changes detected in the juveniles are to be ascribed to the early feeding experience (which occurred several months before the V-challenge) and hence do not represent a direct diet effect as in the early fry. As highlighted in a study dealing with molecular mechanisms involved in nutritional programming in rodents [128], the consistency in the response of key pathways between early developmental and adult stages is considered as a more relevant indicator of nutritional programming than consistency in the direction or magnitude of mRNA levels of individual genes. A follow-up proteomic analysis could be interesting, especially in the liver, to have a better overview of the functional changes in hepatic metabolic processes due to nutritional programming. In this respect, it is encouraging that our results on cholesterol and carbohydrate metabolism compare favourably with proteome data from a previous study in trout [9]. Additionally, candidate enzymatic studies, as in the case of protease regulation and xenobiotic metabolism, in both the 3-week fry and in the juvenile trout liver could help clarify the role of these enzymes in fish fed plant-ingredient rich feed.

Overall, the present molecular dataset, complementary to our previous data on the positive effect of prior plant-diet feeding on its later acceptance (feed intake and feed utilisation efficiency) in trout [34], fortifies the notion that the period of transition from reliance on yolk to exogenous feeding is a critical window for nutritional programming in rainbow trout, and possibly in other teleosts. Persistent molecular changes induced by dietary changes at the same ontogenic first-feeding stage have been observed in the field of hyperglucidic programming in several teleost species, such as trout [25, 26], zebrafish [130] and Siberian sturgeon [131]. Yet, the timing to evaluate the underlying molecular mechanisms deserves further consideration. We analysed the transcriptome changes in trout fry after a 3 week first-feeding exposure, and in liver and brain of juvenile trout at the end of the 25 day V-challenge period in which nutritionally programmed trout exhibit phenotypic enhancement of feed intake and growth. It might, however, be interesting to analyse parallel transcriptomic changes in juvenile trout just before the V-challenge, when both M-fish and V-fish are maintained on the control M-diet. This could be a pertinent sampling window to assess plausible epigenetic modifications of DNA methylation or histone modifications at genes that are nutritionally programmed by the early plant diet-exposure, but exhibit dormant transcriptional responses to the nutritional programming.

## Conclusions

Our results elaborate the transcriptomic changes in trout fry at the first-feeding stage, and in the brain and liver of juvenile trout, accompanying the nutritional programming that enhances feed acceptance of a plant-based diet in rainbow trout. We observe that nutritional programming in the trout brain affects the pathways of sensory perception, synaptic transmission, cognitive processes and neuroendocrine peptides, suggesting the probable acquisition of flavour and feed preference due to early plant-diet exposure. In the liver, changes in pathways mediating intermediary metabolism, xenobiotic metabolism, proteolysis, and cytoskeletal regulation of cell cycle are affected by the prior plant-diet exposure, and some of these changes are congruous with those observed in previous nutrigenomic studies on plant-feeding in cultured fish species. Moreover, nutritional programming appears to reverse the trend in certain hepatic gene expression changes that were previously reported to be affected by plant-diet exposure. Overall, the work in this study highlights the first-feeding stage in trout and possibly other oviparous fish as a critical window for nutritional programming, and provides support for utilizing this strategy to achieve improvements in sustainability of feeding practices in aquaculture.

## Methods

### Experimental design and animal material

The present study aims to gain insight into mechanisms governing the positive effect of an early short term exposure of rainbow trout (*Oncorhynchus mykiss*) fry to a plant-based diet on its future acceptance and utilization, as demonstrated in [34]. Long-term changes in gene expression in brain and liver of juvenile rainbow trout fed diet V (during Challenge V) as a function of the first-feeding diet (diet M or diet V) were therefore analyzed. Details on diet preparation, fish rearing conditions and feeding protocol can be found in [34]. In summary, two experimental diets were prepared: the plant-based diet V (vegetable) contained a blend of protein-rich plant ingredients (wheat gluten, extruded peas, corn gluten meal, soybean meal and white lupin) and a plant oil blend (palm, rapeseed and linseed) as lipid source, whereas diet M (marine) had fishmeal as protein source and fish oil as lipid source. Both diets (Table 1) fulfilled the established nutrient requirements of rainbow trout [4]. The fish selected for the current study is the isogenic heterozygous family C1 (see [34]) of rainbow trout. The applied feeding protocol is based on the concept of nutritional programming in mammals, i.e. the evaluation of a long-term effect due to a specific early-life nutritional event, as applied in other studies with rainbow trout [25, 26]. *O. mykiss* swim-up fry (7 °C water temperature) at first-feeding were fed for 3 weeks either the plant-based diet V or the diet M, after which both groups received diet M during the 7-month intermediate growth phase (Fig. 1a). Both groups (*n* = 3 and 4 replicate tanks for fish of nutritional history V and M, respectively) were then challenged for 25 days by feeding diet V (Fig. 1a) during which voluntary feed intake, growth and feed utilisation efficiency were monitored (16.5 °C water temperature). No difference in initial body weight due to the early feeding experience was noted at the start of the V-challenge (33.6 g, see [34]). Remarkably, fish of nutritional history V compared to M displayed a higher feed intake and growth rate (*P* < 0.05) and a tendency for improved efficiency of feed utilization (*P* = 0.06) during the final V-challenge (Fig. 1), underlining the positive impact of the early diet V feeding history.

**Table 1:** Formulation and composition of diets

Ingredients (g 100 g^−1^ diet)	Diet M	Diet V
Fish oil	8.5	–
Plant oil blend^a^	–	10.3
Fishmeal LT (CP 70 %)	63	–
White lupinseed meal (CP 40 %)	–	5.8
Corn gluten meal (CP 62 %)	–	17.4
Soybean meal (CP 46 %)	–	21.5
Wheat gluten (CP 80 %)	–	25.6
Whole wheat (CP 10 %)	25.4	5.1
Extruded dehulled peas (CP 24 %)	–	3.1
Soy-lecithin	–	2.0
L-Arginine	–	1.0
L-Lysine	–	1.5
CaHPO4.2H20 (18%P)	–	3.6
Mineral and vitamin premix^b^	3.0	3.0
Analysed composition		
Dry matter (DM, % diet)	93.3	92.4
Crude protein (% DM)	52.1	50.5
Crude fat (% DM)	17.9	17.0
Gross energy (kJ g^−1^ DM)	22.3	22.3

Reproduced from [34]. Formulation, approximate crude protein (CP) levels of ingredients and analysed composition of the experimental diets M (fishmeal and fish oil-based) and V (all fishmeal and fish oil replaced by plant protein and plant oil sources). ^a^Consisting of (% blend): rapeseed oil (50), palm oil (30), linseed oil (20). ^b^INRA UPAE, 78352 Jouy en Josas, France

### RNA isolation

As indicated on Fig. 1, samples were taken at the end of the 3-week early first-feeding exposure (whole fry, overnight fasted) and at the end of the V-challenge (8 h after the last meal) where liver and whole brain were carefully dissected, instantly snap-frozen in liquid nitrogen and stored at −80 °C prior to RNA extraction. The first sampling (fry) thus assesses the immediate short-term effect on the profile of gene expression, i.e. effect diet V vs. M, as opposed to the second sampling (juvenile tissue) used to evaluate potential long-term effects related to the early life exposure, i.e. effect nutritional history M vs. V. Total RNA was extracted from the trout swim-up fry, brain or liver by homogenization in 1–2 ml TRIzol (Invitrogen, Carlsbad, CA, USA) reagent following the manufacturer’s instructions. Removal of genomic DNA from RNA samples was performed using RNeasy mini spin columns (Qiagen) through on-filter DNase digestion according to the manufacturers recommendations. Total RNA was quantified using spectrophotometry based on absorbance at 260 nm (Nanodrop ND1000, LabTech) and the integrity of the RNA was determined by electrophoresis (Agilent Bioanalyser 2100) and samples with RNA integrity number (RIN) ≥ 8 were used for microarray analysis.

### Microarray platform

Microarray experiments were performed on an Agilent-based microarray platform with 8 X 60 K probes per slide (Gene Expression Omnibus; GEO platform record: GPL19030). This platform is based on a 37 K high density rainbow trout oligonucleotide microarray resource designed by Yao and colleagues [132], as previously employed at Laboratoire de Physiologie et Génomique des Poissons (LPGP, Institut National de la Recherche Agronomique, Rennes, France) [133] and has been enriched with oligonucleotides designed utilizing recent NGS data from trout [134]. The microarray gene annotations were reanalysed by Sigenae (Institut National de la Recherche Agronomique, Toulouse, France). Microarray data sets have been submitted to the GEO-NCBI with the accession number GSE60010.

They may be accessed at http://www.ncbi.nlm.nih.gov/geo/query/acc.cgi?token=sfafokqmtlevbop&acc=GSE60010.

### Microarray hybridization, data acquisition and analysis

Distinct RNA samples from trout swim-up fry exposed to diet M (*n* = 4 fry) or diet V (*n* = 3 fry) for 3 weeks, and whole brain (*n* = 4 samples) and whole liver (*n* = 4 samples) of juvenile trout with nutritional history of diet M or diet V after the 25 day V-challenge were used for labelling and hybridisation. For each sample, 150 ng of total RNA was amplified and labelled using Cy3-CTP according to the manufacturer’s instructions (Agilent). Briefly, RNA was first reverse transcribed, using a polyDT T7 primer, Cy3 was then incorporated by a T7 polymerase mediated transcription and excess dye was removed using a RNeasy kit (Qiagen). The level of dye incorporation was evaluated using a spectrophotometer (Nanodrop ND1000, LabTech). Yield (>0.825 μg cRNA) and specific activity (>6pmol of Cy3 per μg of cRNA) of the Cy3-cRNA produced were checked with the Nanodrop ND1000 (LabTech). 600 ng of Cy3-cRNA was then fragmented in the appropriate buffer (Agilent) for 30 min at 60 °C and hybridized on a sub-array. Hybridisation was performed in a microarray hybridization oven (Agilent) for 17 h at 65 °C. The slides were then rinsed in gene expression wash buffers 1 and 2 (Agilent) and scanned with an Agilent scanner (Agilent DNA Microarray Scanner, Agilent Technologies, Massy, France) using the standard parameters for a gene expression 8x60K oligoarray (3 μm and 20 bits). Data were then obtained with the Agilent Feature Extraction software (10.7.1.1) according to the appropriate GE protocol (GE1_107_Sep09). Arrays were normalised using scale normalization with GeneSpring software (version 12.6.1). We performed a 2- way ANOVA on log 2-transformed normalized data, using a *p*-value with Benjamini-Hochberg correction [False Discovery Rate; FDR] ≤ 0.05 and fold-change ≥ 1.5, to determine the probes that are differentially expressed by early V vs M feeding in 3-week fry and in juvenile brain (further called ‘3-week + Brain’) and, similarly, a 2-way ANOVA to determine the probes that are differentially expressed by early V vs M feeding in both 3-week fry and in juvenile liver (further called ‘3-week + Liver’). ANOVA were performed using Statistica 8.0 (StatSoft, OK, USA). For clustering analysis, data were log transformed, median-centred and an average linkage clustering was carried out using CLUSTER software [135]. The results were visualised using TREEVIEW [136].

### Gene ontology analysis

Gene ontology (GO) analysis was performed using the functional annotation cluster and chart tools from the DAVID (Database for Annotation, Visualization and Integrated Discovery) bioinformatics resource [137, 138]. Briefly, the Uniprot accession ID’s of the mRNA probes differentially expressed by nutritional history (Additional file 1, 3-week + Brain; 3-week + Liver) were used as input against a background Uniprot accession ID’s of the entire 60 K array probe list and processed with the functional annotation cluster and chart tools from the DAVID bioinformatics resource. The resulting data was used as input in the DAVID Enrichment Map plugin from the Bader Lab [139, 140] in the Cytoscape network visualization tool (version 2.8.3) [141–143].

### Pathway analysis

Selected mRNA probes differentially expressed by nutritional history (3 week + Brain; 3-week + Liver) and those assigned to be part of a significantly affected GO process (Additional files 5 and 6) were used as inputs in the GeneMania pathway analysis tool [35–37] using the inbuilt *Homo sapiens* pathway interaction database as background and processed according to the default settings in the GeneMania tool. The pathway maps generated by GeneMania are outlined in Fig. 5 and Additional files 3 and 4.

### Real time PCR

Gene expression levels were determined by real-time RT-PCR using the same RNA samples (*n* = 4) that were also employed in the labelling and hybridisation for microarray analysis. Total RNA (1 μg) samples from trout swim-up fry exposed to diet M (*n* = 4 fry) or diet V (*n* = 4 fry) for 3 weeks, and whole brain (*n* = 4 samples) and whole liver (*n* = 4 samples) of juvenile trout with nutritional history of diet M or diet V after the 25 day V-challenge were used to reverse transcribed to cDNA with the Superscript III RT kit (Invitrogen, Carlsbad, CA, USA) using oligo dT primers. Real-time PCR was performed in the iCycler MyiQ Real-Time PCR detection system (Bio-Rad, Hercules, CA, USA). The genes chosen for the swim-up fry were keratin 13 (K13), Lissencephaly-1 homolog B (pafah1b1b), purpurin (rbp4l) and chymotrypsin B (Ctrb1). The genes chosen for the brain were Glucagon-2 (gcg2), purpurin (rbp4l), somatolactin (SL), Recoverin (Rcvrn) and cholecystokinin-Thr (CCK-T). The genes chosen for the liver were chymotrypsin B (Ctrb1), keratin 13 (K13) and cytosolic sulfotransferase 3 (ST1S3). Quantitative PCR analyses for gene expressions were performed on 10 μl of the RT reaction mixture using the iQ SYBR Green Supermix (Bio-Rad). The total volume of the PCR reaction was 25 μl containing 200 nM of each primer. The primers used are outlined in Additional file 8. Thermal cycling was initiated with the incubation at 95 °C for 90 s for hot-start iTaq DNA polymerase activation. Forty steps of PCR were performed, each one consisting of heating at 95 °C for 20 s for denaturing, and at 60 °C for 30 s for annealing and extension. Following the final cycle of the PCR, melting curves were systematically monitored (with a gradient of 0 · 5 °C per 10 s from 55 to 95 °C) to ensure that only one fragment was amplified. Samples without RT and samples without RNA were run for each reaction as negative controls. The mRNA expression levels were normalized using actin as the reference gene and the fold-change obtained by normalizing to the respective expression in fish fed diet M (M-diet or history of M-diet). The significance of mRNA expression between groups was determined using an unpaired *t*-test (Mann-Whitney test; *p* ≤ 0.05).

## Additional files

Additional file 1: List of mRNA probes differentially expressed based on nutritional history in the brain and liver. The lists of 1787 (3-week + Brain) and of 924 (3-week + Liver) differentially expressed mRNA probes (fold change ≥ 1.5 and p [nutritional history] ≤ 0.05), both at the end of the early exposure (Fig. 1; 3-week, diet V vs. diet M) and in the brain or liver of juvenile trout at the end of the V-challenge (Fig. 1; V-his vs. M-his). The list of 159 probes with overlapping significant expression profiles in both brain and liver comparisons is shown in a separate worksheet. Probe names highlighted in yellow were selected for RT-PCR confirmation. (XLSX 216 kb) Additional file 2: Heat map of hierarchical clustering of differentially expressed mRNA probes by nutritional history. The horizontal dendrogram represents the correlation distances between gene expression levels. Each row represents the expression of a single mRNA probe and each column represents a single sample as follows: (a) and (b), trout swim-up fry exposed to diet M (columns 1–4) or diet V (columns 5–7) for 3 weeks; (a) Brain and (b) Liver, of juvenile trout with nutritional history of diet M (M-his, columns 8–11) or diet V (V-his, columns 12–15) after the 25 day V-challenge with plant based-diet. The inset box (c) gives a legend for expression levels (log [fold change]), with red representing high levels of expression and green representing low levels of expression. (PPTX 137 kb) Additional file 3: Pathways significantly enriched based on early nutritional history in the brain. The mRNA probes (see Additional file 5) that were assigned to be part of the pathways homocysteine and methionine metabolism; and neuroendocrine peptides were used as input in the GeneMania pathway analysis tool [35–37] to generate networks. The functions legend (nodes) represents the sub-network of the mRNA probes and the network legend (lines) represents the relationship between the genes (see methods). (PPTX 1177 kb) Additional file 4: Pathways significantly enriched based on early nutritional history in the liver. The mRNA probes (see Additional file 6) that were assigned to be part of the pathways intermediary metabolism; oxidation-reduction; zymogens; peptidyl-prolyl-isomerases; cytoskeleton; and cell cycle were used as input in the GeneMania pathway analysis tool [35–37] to generate networks. The functions legend (nodes) represents the sub-network of the mRNA probes and the network legend (lines) represents the relationship between the genes (see Methods). (PPTX 6035 kb) Additional file 5: List of probes in pathways significantly enriched based on early nutritional history in the brain. The list of differentially expressed mRNA probes comprising the pathways neurological system process; homocysteine and methionine metabolism; and neuroendocrine peptides that are significantly enriched based on early nutritional history in the brain are outlined in separate worksheets. Probe names highlighted in yellow were selected for RT-PCR confirmation. (XLSX 18 kb) Additional file 6: List of probes in pathways significantly enriched based on early nutritional history in the liver. The list of differentially expressed mRNA probes comprising the pathways intermediary metabolism; oxidation-reduction; zymogens; peptidyl-prolyl-isomerases; cytoskeleton; and cell cycle that are significantly enriched based on early nutritional history in the liver are outlined in separate worksheets. Probe names highlighted in yellow were selected for RT-PCR confirmation. (XLSX 28 kb) Additional file 7: Confirmation by real-time RT-PCR of selected genes differentially expressed in trout swim-up fry (a), juvenile brain (b) and liver (c) based on early exposure to plant-based diet. Bars represent mean ± standard deviation of four fish, asterisks indicate significant (*p* < 0.05) differences. The fold change as identified by microarray analysis is outlined in the accompanying table. The genes chosen for the swim-up fry were keratin 13 (K13), Lissencephaly-1 homolog B (pafah1b1b), purpurin (rbp4l) and chymotrypsin B (Ctrb1). The genes chosen for the brain were Glucagon-2 (gcg2), purpurin (rbp4l), somatolactin (SL), Recoverin (Rcvrn) and cholecystokinin-Thr (CCK-T). The genes chosen for the liver were chymotrypsin B (Ctrb1), keratin 13 (K13) and cytosolic sulfotransferase 3 (ST1S3). The data from microarray experiments for probe expression of the corresponding genes is included in the table below the graphs. (PPTX 281 kb) Additional file 8: List of primers used in real-time RT-PCR analysis for confirmation of differential expression of selected genes identified by the microarray analysis. (XLSX 11 kb)
