# Supplementary material for: Molecular pathways associated with the nutritional programming of plant-based diet acceptance in rainbow trout following an early feeding exposure
Source: BMC Genomics. 2016 Jun 13;17:449. doi: 10.1186/s12864-016-2804-1 (PMC4907080; doi:10.1186/s12864-016-2804-1)
Supplement: Additional file 2: — Heat map of hierarchical clustering of differentially expressed mRNA probes by nutritional history. The horizontal dendrogram represents the correlation distances between gene expression levels. Each row represents the expression of a single mRNA probe and each column represents a single sample as follows: (a) and (b), trout swim-up fry exposed to diet M (columns 1–4) or diet V (columns 5–7) for 3 weeks; (a) Brain and (b) Liver, of juvenile trout with nutritional history of diet M (M-his, columns 8–11) or diet V (V-his, columns 12–15) after the 25 day V-challenge with plant based-diet. The inset box (c) gives a legend for expression levels (log [fold change]), with red representing high levels of expression and green representing low levels of expression. (PPTX 137 kb) [file 12864_2016_2804_MOESM2_ESM.pptx]

## Slide 1
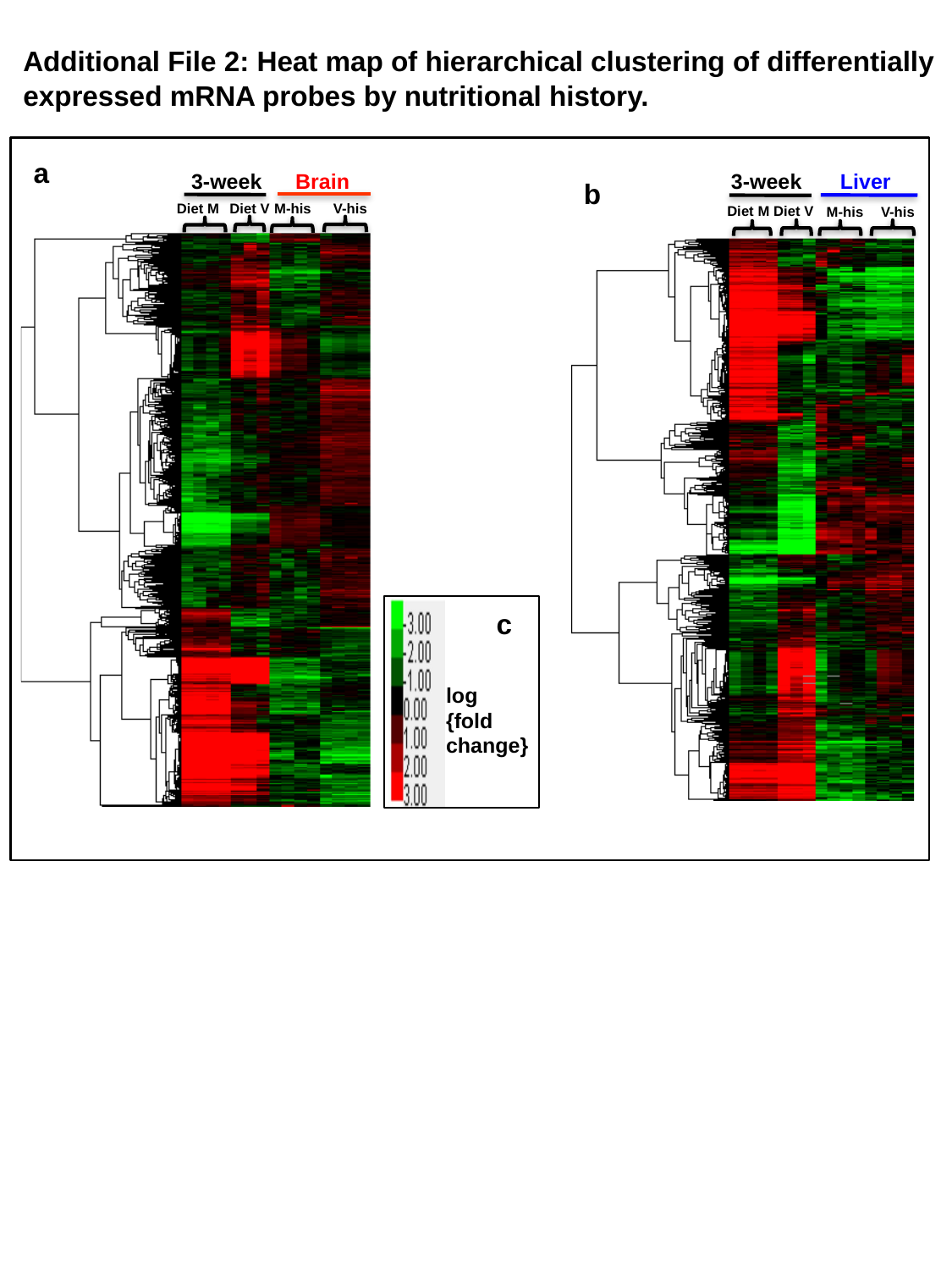

Additional File 2: Heat map of hierarchical clustering of differentially expressed mRNA probes by nutritional history.
a
3-week
Brain
Diet M
Diet V
M-his
V-his
3-week
Liver
Diet V
Diet M
M-his
V-his
b
c
log
{fold change}
