# Supplementary material for: Molecular pathways associated with the nutritional programming of plant-based diet acceptance in rainbow trout following an early feeding exposure
Source: BMC Genomics. 2016 Jun 13;17:449. doi: 10.1186/s12864-016-2804-1 (PMC4907080; doi:10.1186/s12864-016-2804-1)
Supplement: Additional file 3: — Pathways significantly enriched based on early nutritional history in the brain. The mRNA probes (see Additional file 5) that were assigned to be part of the pathways homocysteine and methionine metabolism; and neuroendocrine peptides were used as input in the GeneMania pathway analysis tool [35–37] to generate networks. The functions legend (nodes) represents the sub-network of the mRNA probes and the network legend (lines) represents the relationship between the genes (see methods). (PPTX 1177 kb) [file 12864_2016_2804_MOESM3_ESM.pptx]

## Slide 1
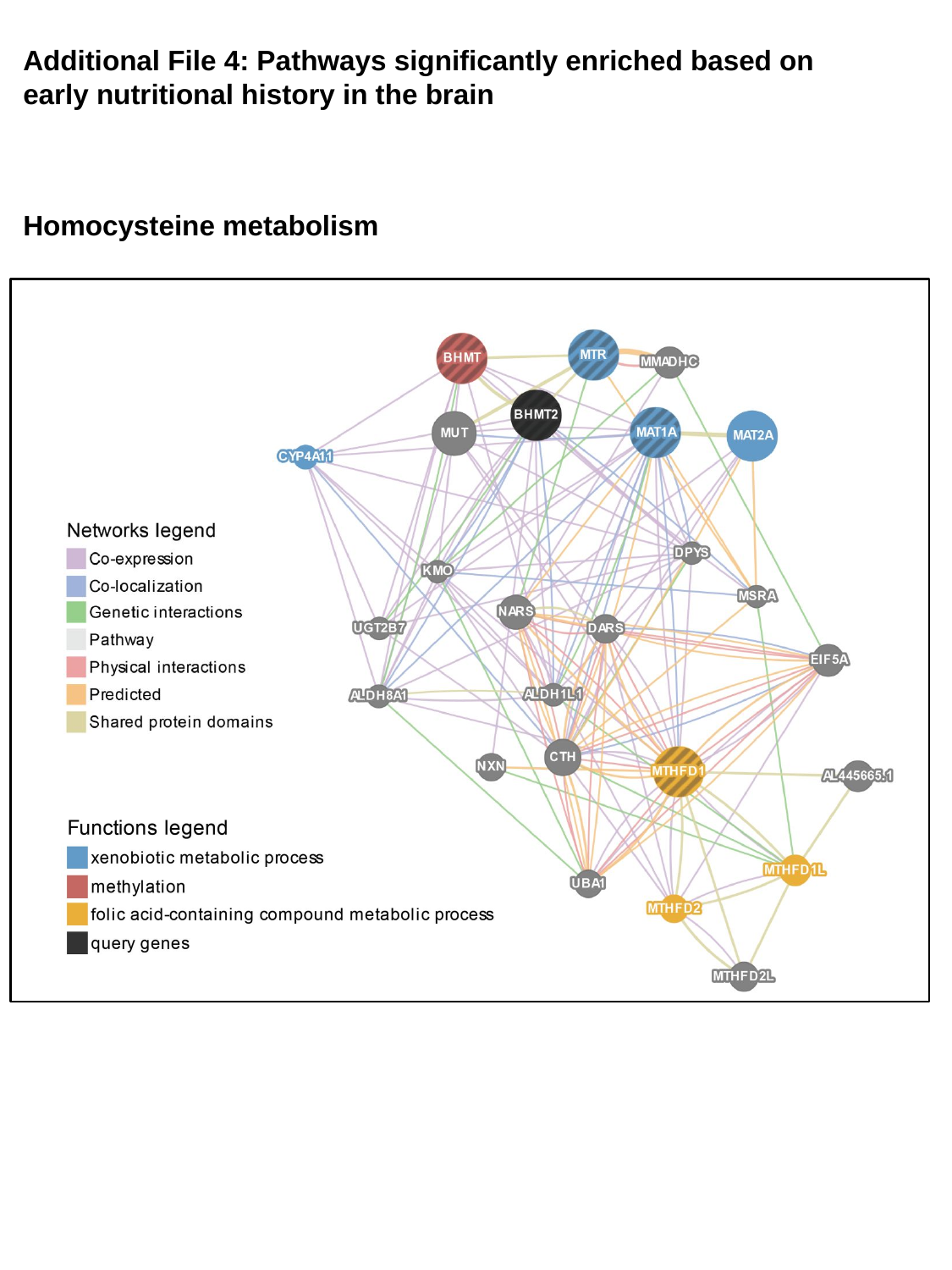

Additional File 4: Pathways significantly enriched based on early nutritional history in the brain
Homocysteine metabolism

## Slide 2
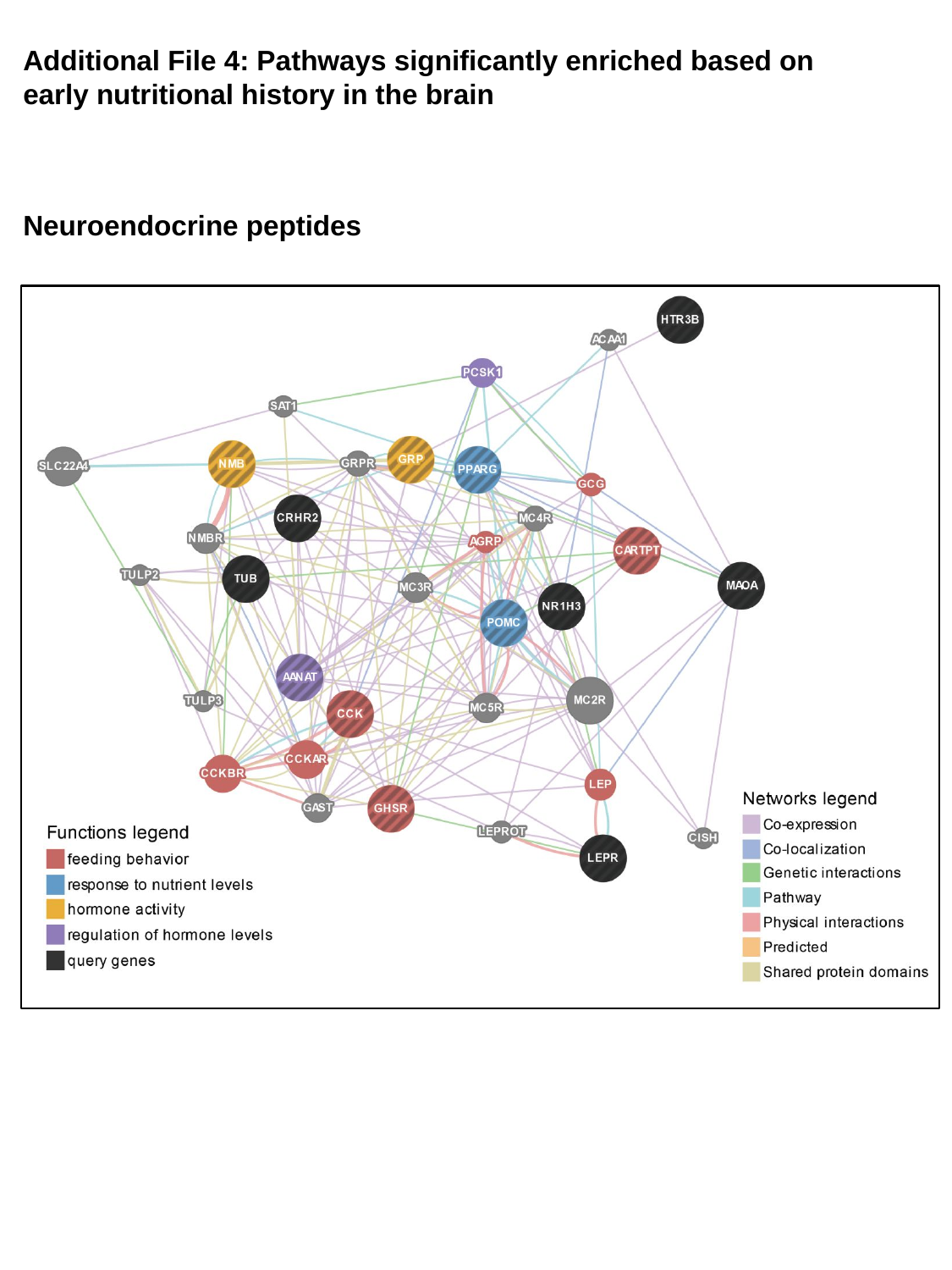

Additional File 4: Pathways significantly enriched based on early nutritional history in the brain
Neuroendocrine peptides
