# Supplementary material for: Molecular pathways associated with the nutritional programming of plant-based diet acceptance in rainbow trout following an early feeding exposure
Source: BMC Genomics. 2016 Jun 13;17:449. doi: 10.1186/s12864-016-2804-1 (PMC4907080; doi:10.1186/s12864-016-2804-1)
Supplement: Additional file 4: — Pathways significantly enriched based on early nutritional history in the liver. The mRNA probes (see Additional file 6) that were assigned to be part of the pathways intermediary metabolism; oxidation-reduction; zymogens; peptidyl-prolyl-isomerases; cytoskeleton; and cell cycle were used as input in the GeneMania pathway analysis tool [35–37] to generate networks. The functions legend (nodes) represents the sub-network of the mRNA probes and the network legend (lines) represents the relationship between the genes (see Methods). (PPTX 6035 kb) [file 12864_2016_2804_MOESM4_ESM.pptx]

## Slide 1
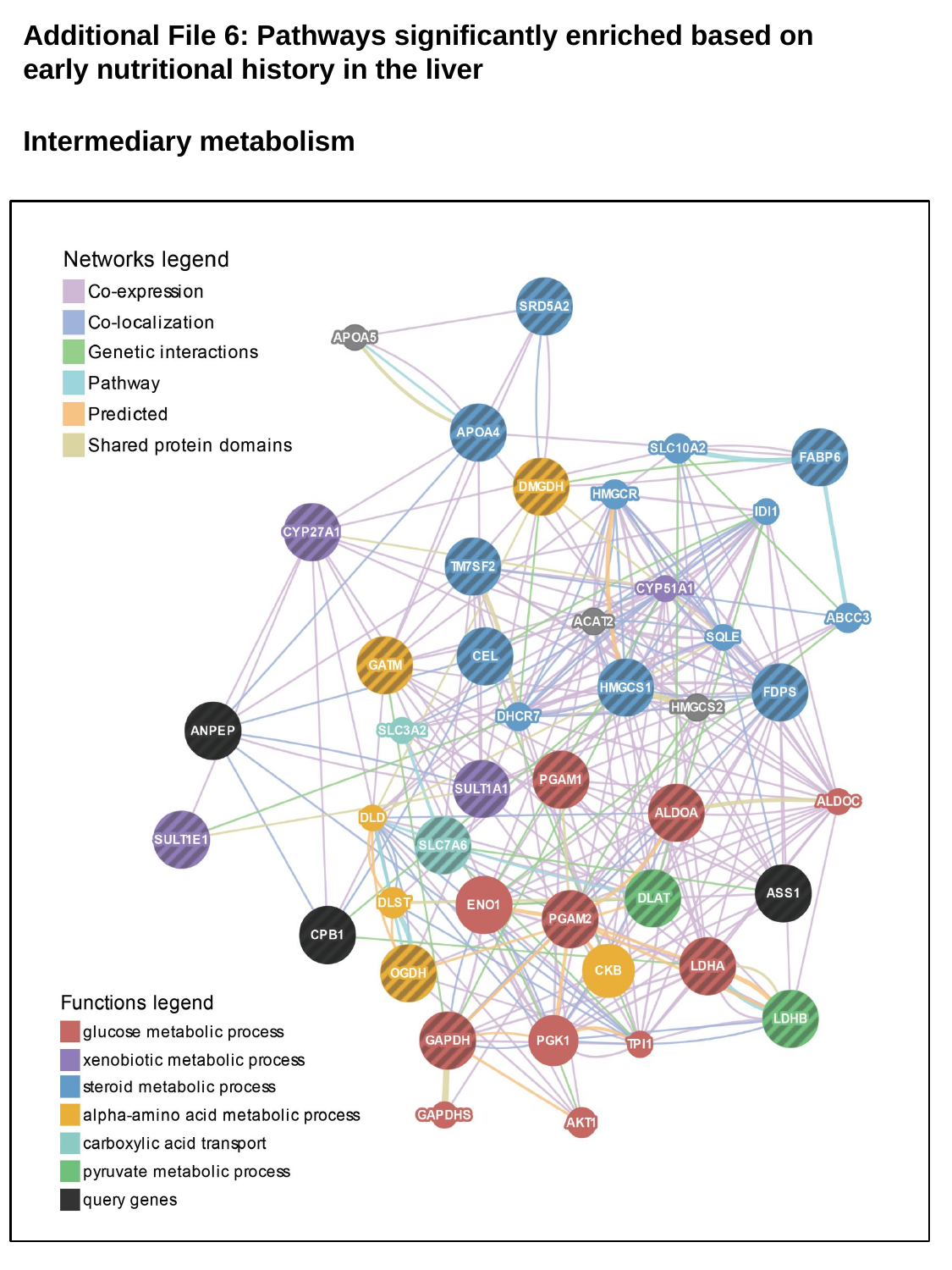

Additional File 6: Pathways significantly enriched based on early nutritional history in the liver
Intermediary metabolism

## Slide 2
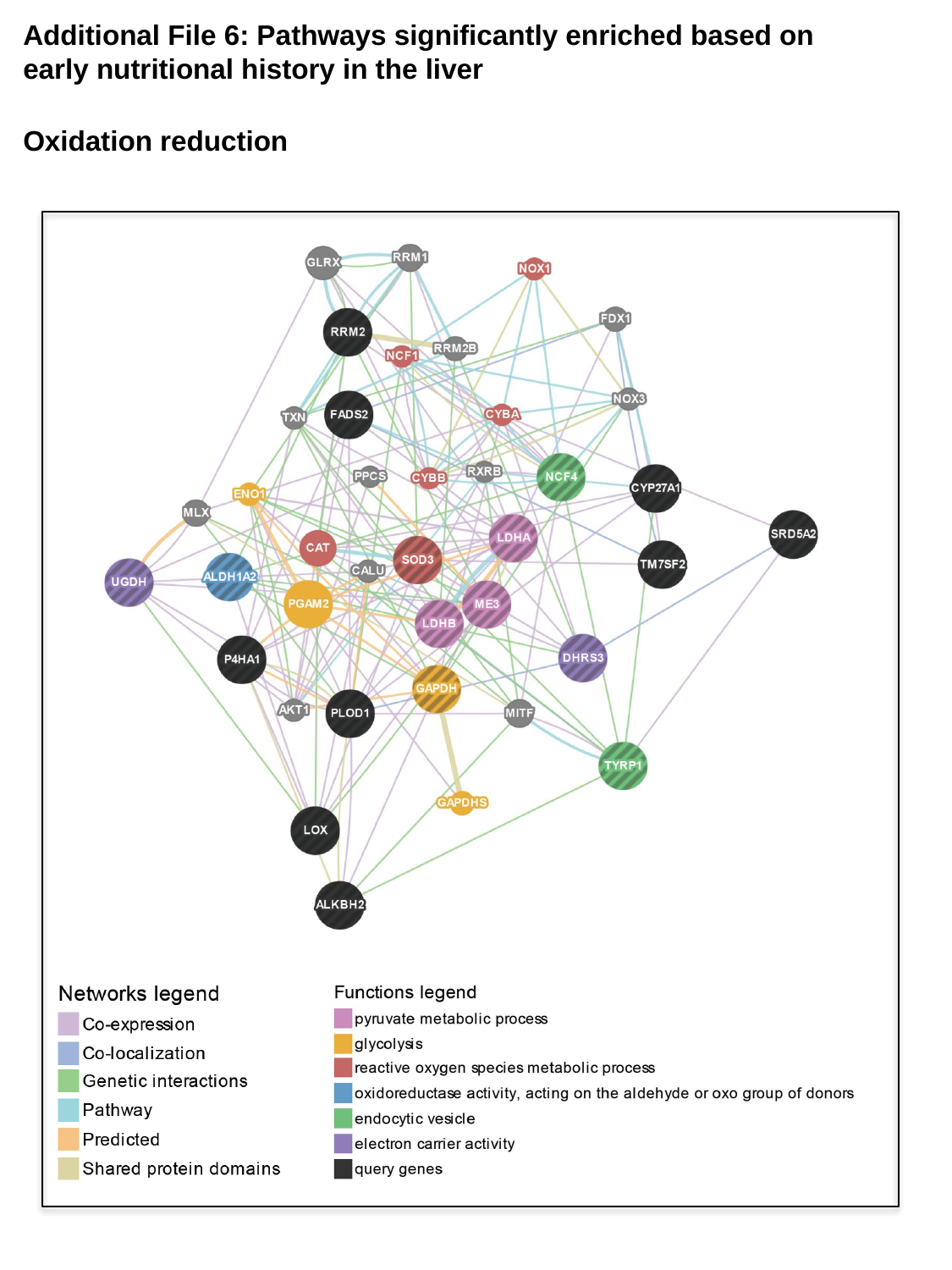

Additional File 6: Pathways significantly enriched based on early nutritional history in the liver
Oxidation reduction

## Slide 3
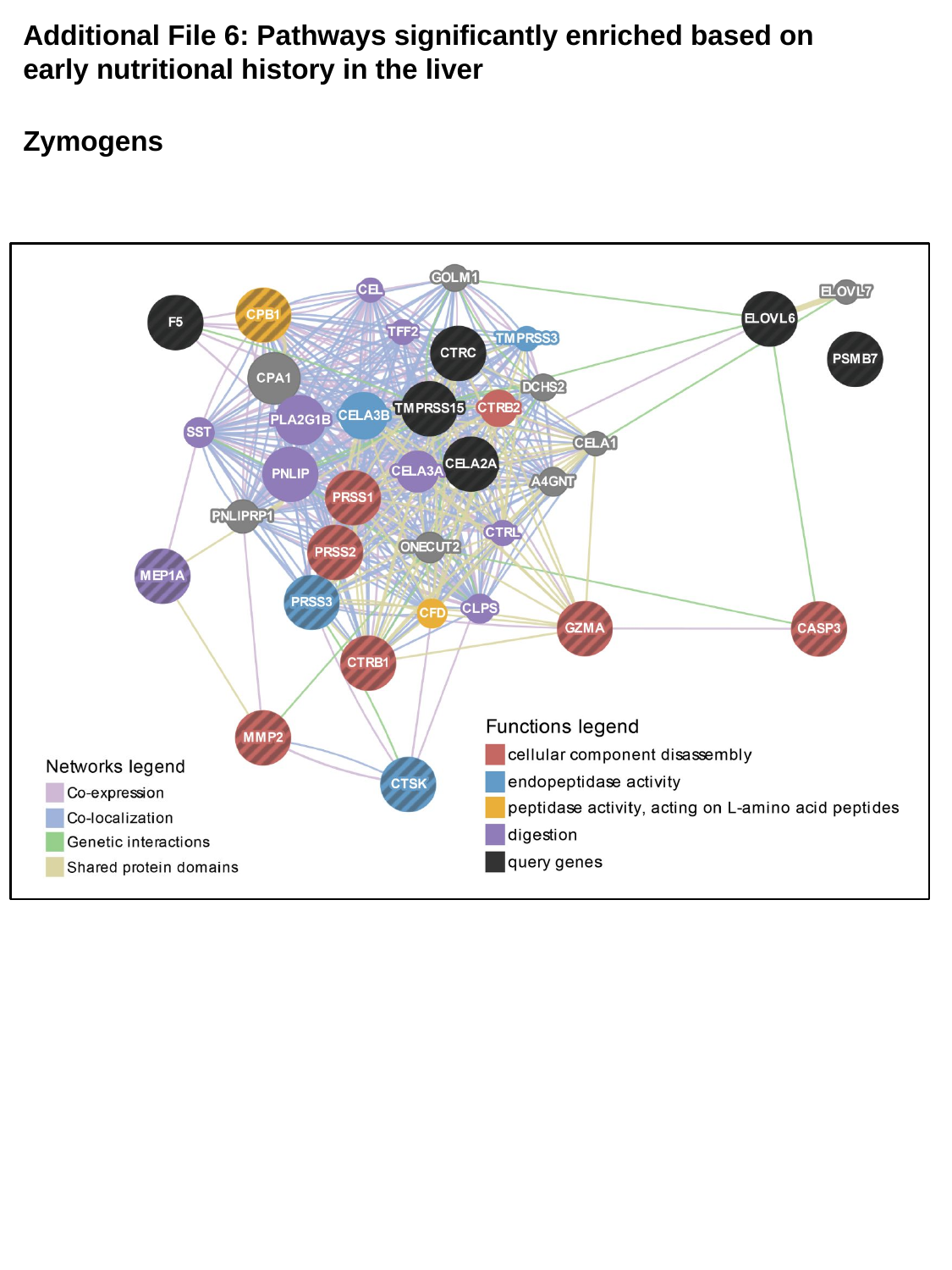

Additional File 6: Pathways significantly enriched based on early nutritional history in the liver
Zymogens

## Slide 4
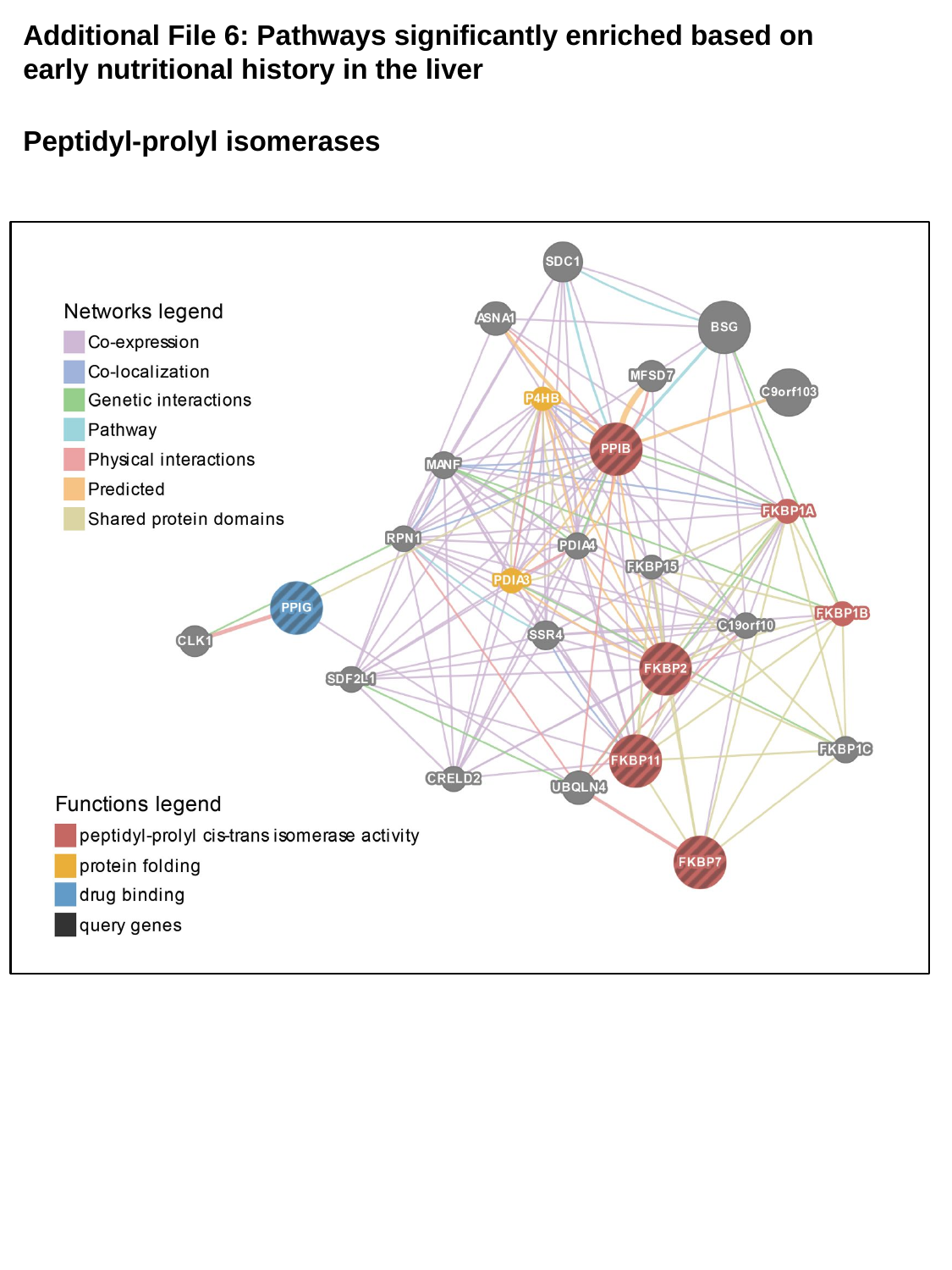

Additional File 6: Pathways significantly enriched based on early nutritional history in the liver
Peptidyl-prolyl isomerases

## Slide 5
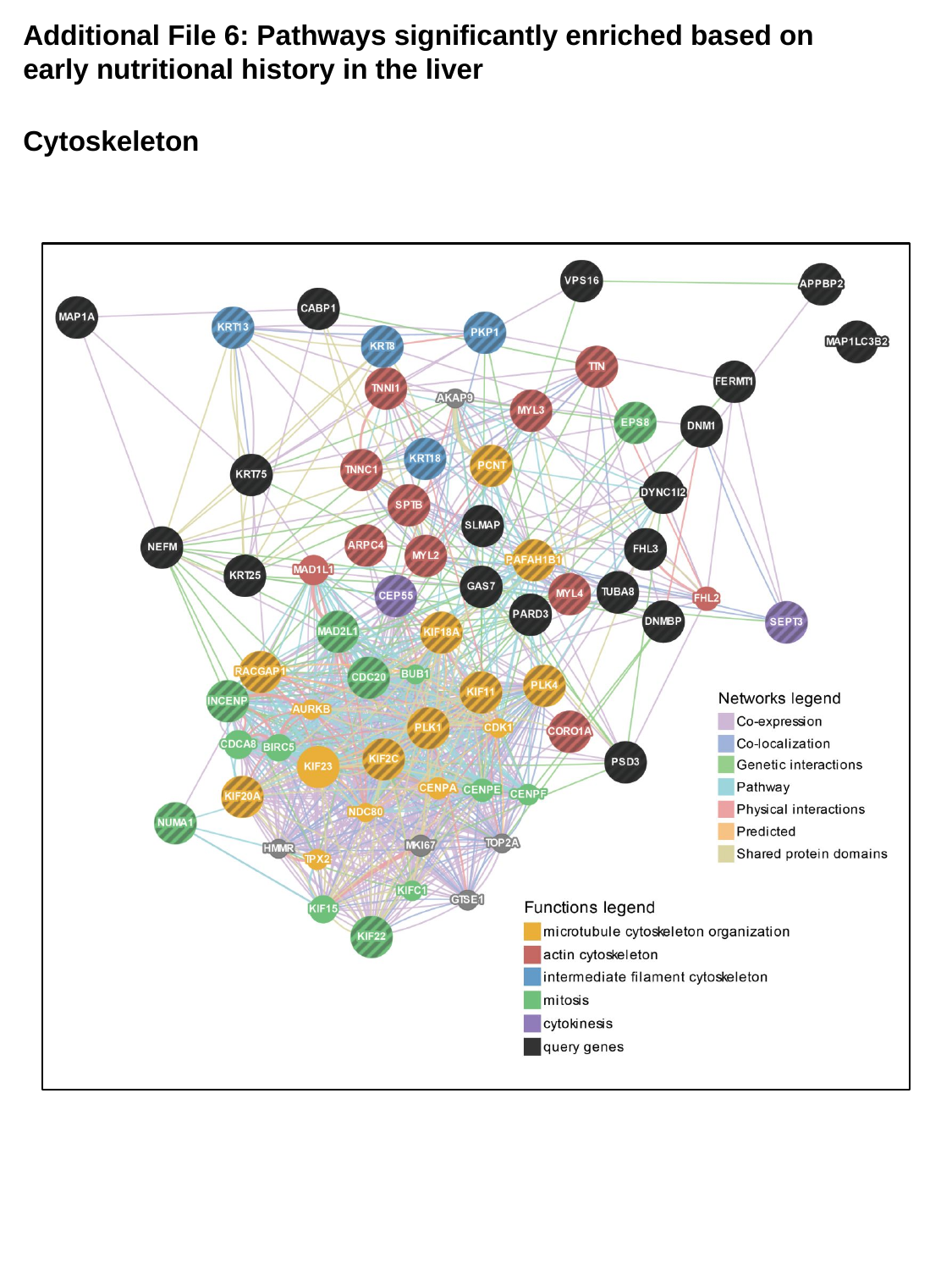

Additional File 6: Pathways significantly enriched based on early nutritional history in the liver
Cytoskeleton

## Slide 6
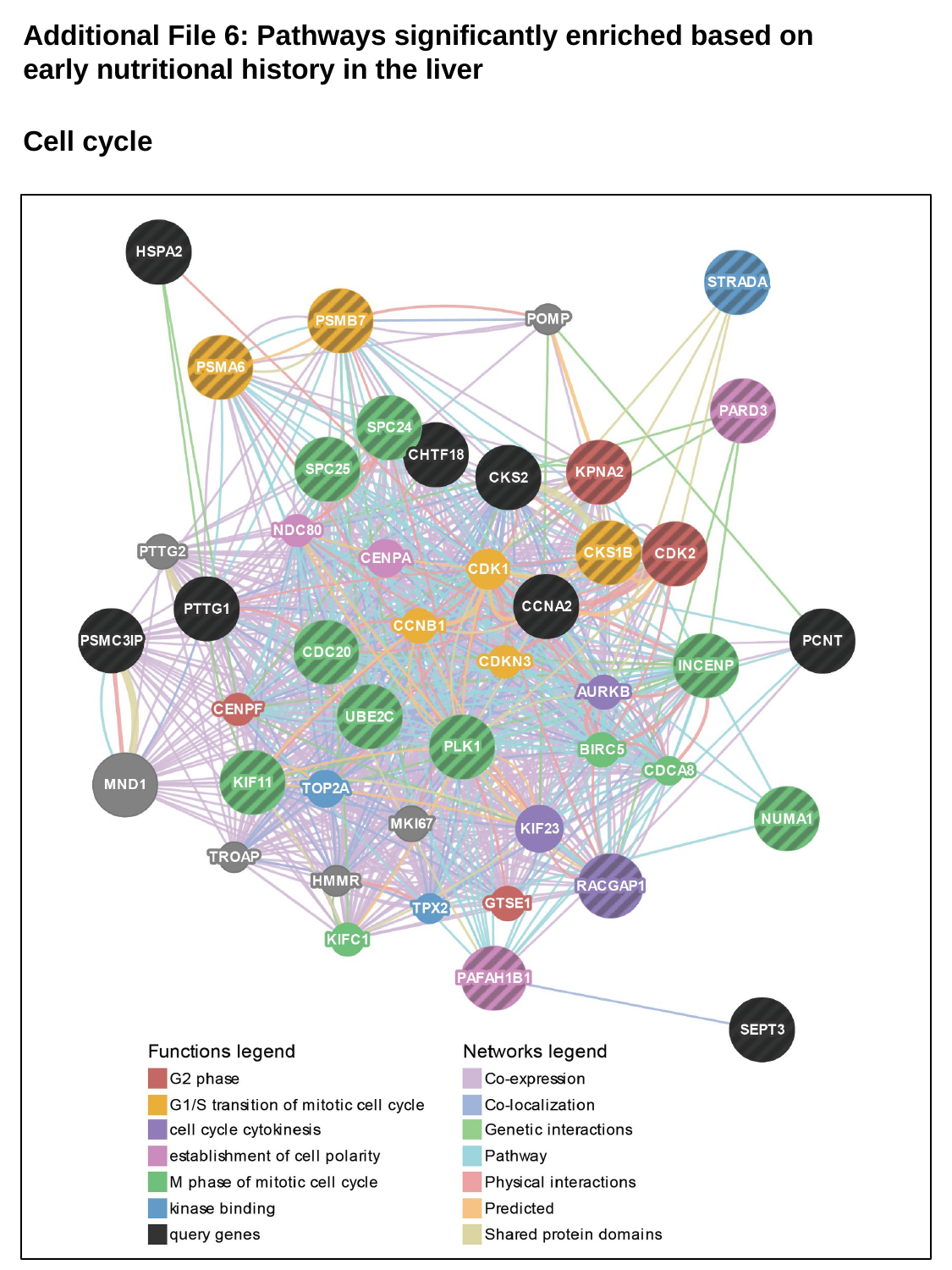

Additional File 6: Pathways significantly enriched based on early nutritional history in the liver
Cell cycle
