# Supplementary material for: Molecular pathways associated with the nutritional programming of plant-based diet acceptance in rainbow trout following an early feeding exposure
Source: BMC Genomics. 2016 Jun 13;17:449. doi: 10.1186/s12864-016-2804-1 (PMC4907080; doi:10.1186/s12864-016-2804-1)
Supplement: Additional file 7: — Confirmation by real-time RT-PCR of selected genes differentially expressed in trout swim-up fry (a), juvenile brain (b) and liver (c) based on early exposure to plant-based diet. Bars represent mean ± standard deviation of four fish, asterisks indicate significant (p < 0.05) differences. The fold change as identified by microarray analysis is outlined in the accompanying table. The genes chosen for the swim-up fry were keratin 13 (K13), Lissencephaly-1 homolog B (pafah1b1b), purpurin (rbp4l) and chymotrypsin B (Ctrb1). The genes chosen for the brain were Glucagon-2 (gcg2), purpurin (rbp4l), somatolactin (SL), Recoverin (Rcvrn) and cholecystokinin-Thr (CCK-T). The genes chosen for the liver were chymotrypsin B (Ctrb1), keratin 13 (K13) and cytosolic sulfotransferase 3 (ST1S3). The data from microarray experiments for probe expression of the corresponding genes is included in the table below the graphs. (PPTX 281 kb) [file 12864_2016_2804_MOESM7_ESM.pptx]

## Slide 1
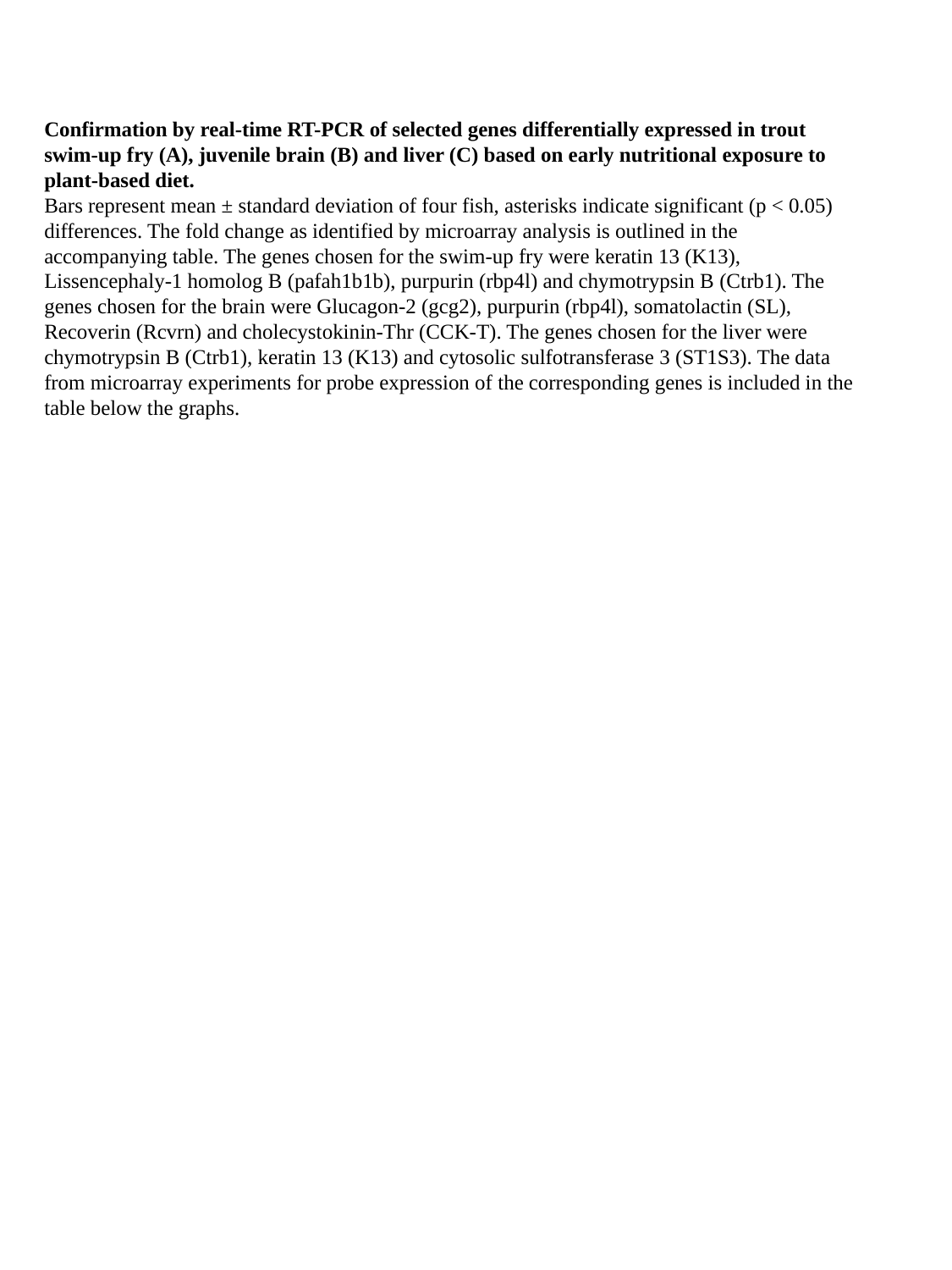

Confirmation by real-time RT-PCR of selected genes differentially expressed in trout swim-up fry (A), juvenile brain (B) and liver (C) based on early nutritional exposure to plant-based diet.
Bars represent mean ± standard deviation of four fish, asterisks indicate significant (p < 0.05) differences. The fold change as identified by microarray analysis is outlined in the accompanying table. The genes chosen for the swim-up fry were keratin 13 (K13), Lissencephaly-1 homolog B (pafah1b1b), purpurin (rbp4l) and chymotrypsin B (Ctrb1). The genes chosen for the brain were Glucagon-2 (gcg2), purpurin (rbp4l), somatolactin (SL), Recoverin (Rcvrn) and cholecystokinin-Thr (CCK-T). The genes chosen for the liver were chymotrypsin B (Ctrb1), keratin 13 (K13) and cytosolic sulfotransferase 3 (ST1S3). The data from microarray experiments for probe expression of the corresponding genes is included in the table below the graphs.

## Slide 2
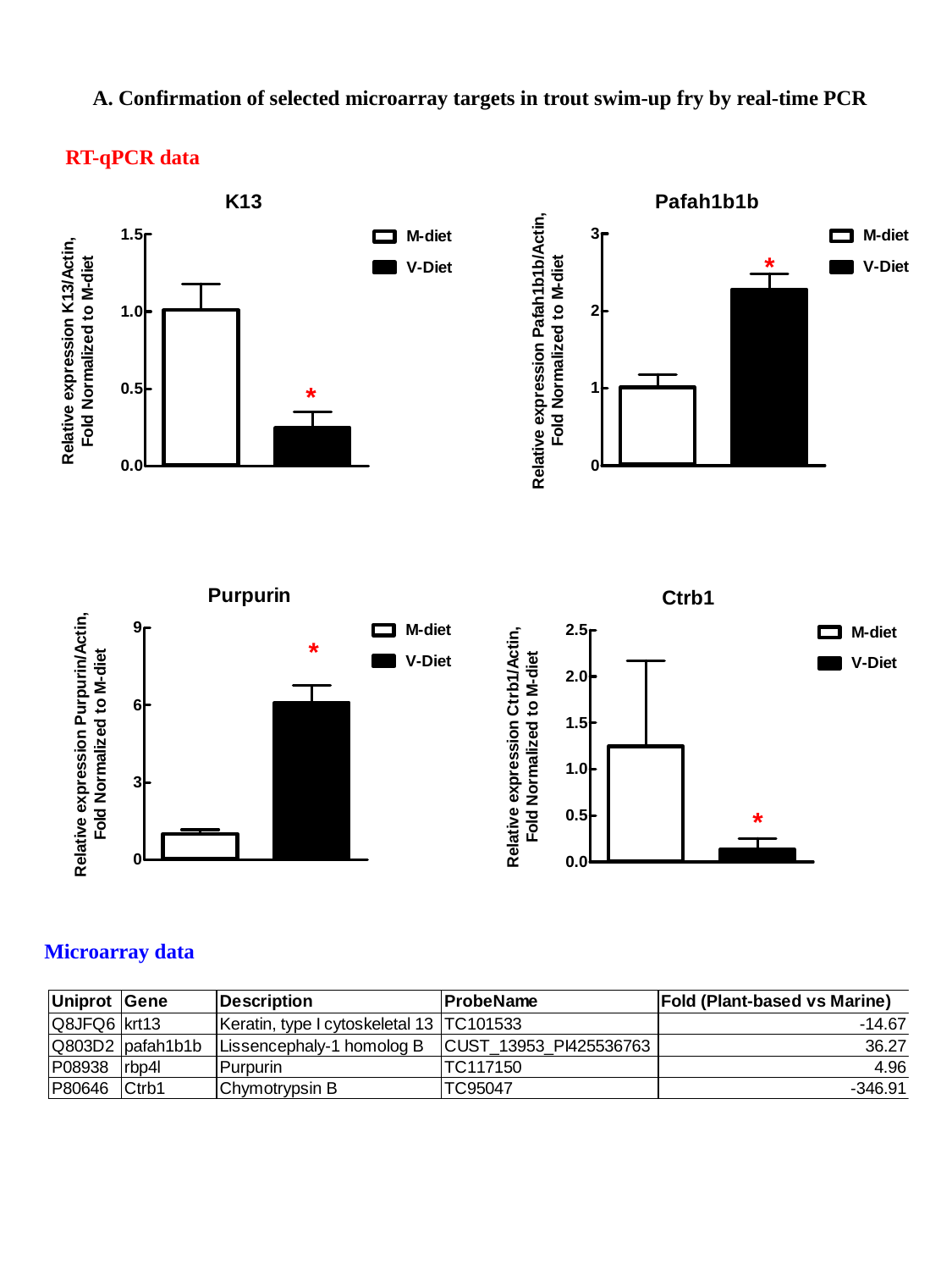

A. Confirmation of selected microarray targets in trout swim-up fry by real-time PCR
RT-qPCR data
Microarray data

## Slide 3
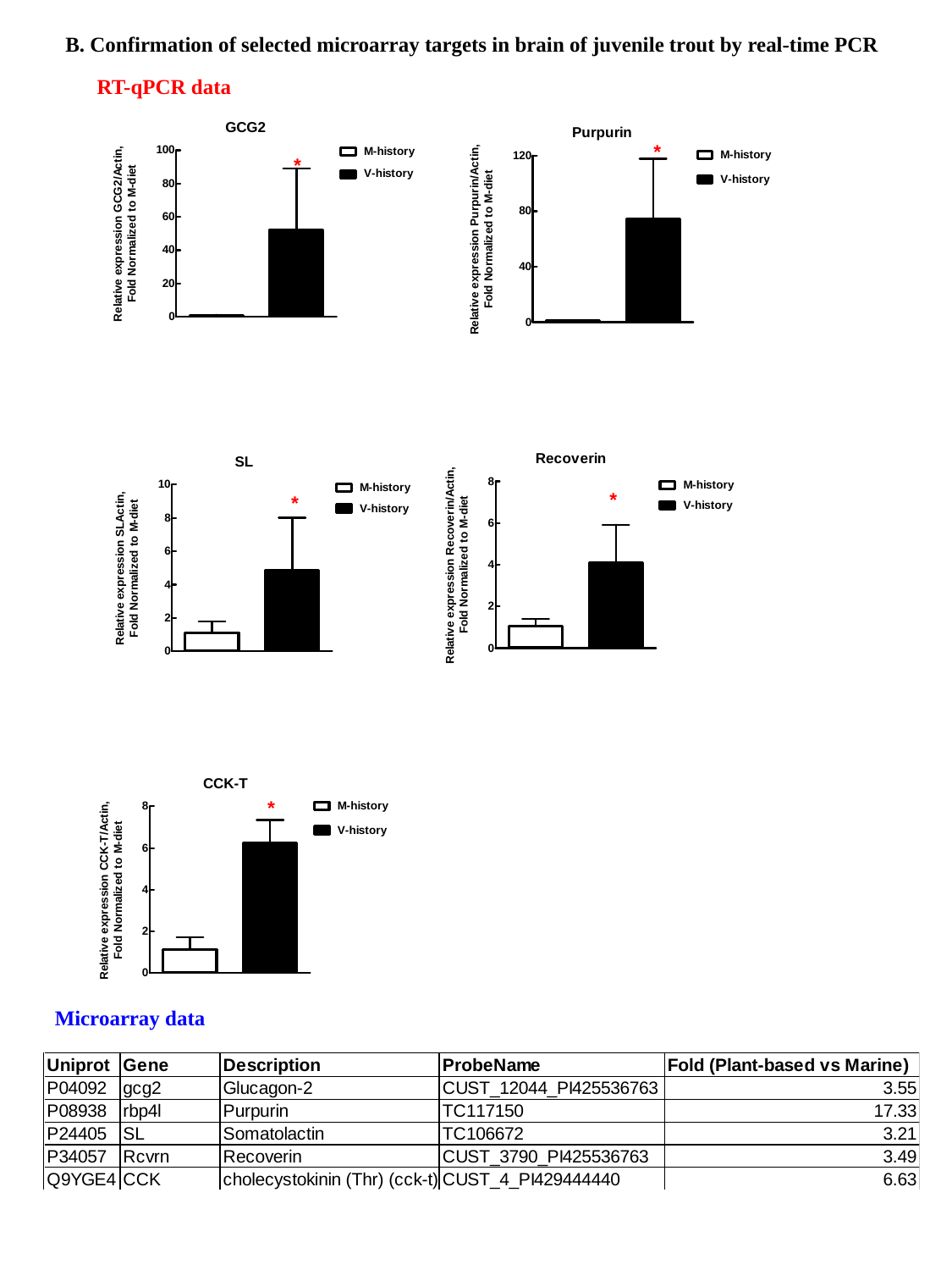

B. Confirmation of selected microarray targets in brain of juvenile trout by real-time PCR
RT-qPCR data
Microarray data

## Slide 4
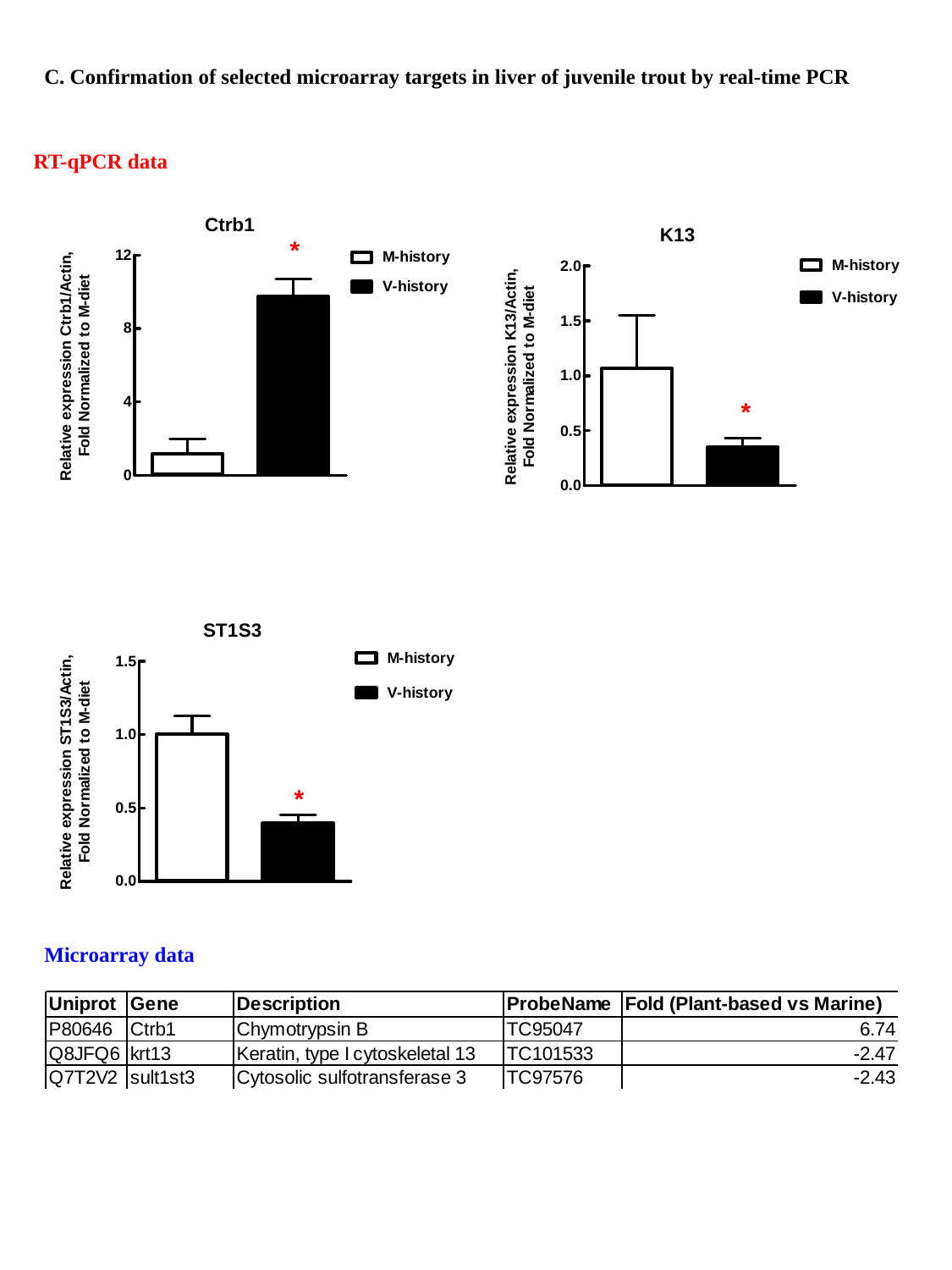

C. Confirmation of selected microarray targets in liver of juvenile trout by real-time PCR
RT-qPCR data
Microarray data
